# Phylogeographic Pattern of *Sargassum hemiphyllum* var. *chinense* (Phaeophyceae, Ochrophyta) in Chinese Coastal Waters

**DOI:** 10.3390/plants14091269

**Published:** 2025-04-22

**Authors:** Zepan Chen, Weizhou Chen, Hong Du

**Affiliations:** 1 Marine Science Institute, Shantou University, Shantou 515063, China; zepan@stu.edu.cn (Z.C.); hdu@stu.edu.cn (H.D.); 2 Guangdong Provincial Key Laboratory of Marine Biotechnology, Shantou University, Shantou 515063, China

**Keywords:** *Sargassum hemiphyllum* var. *chinense*, phylogeography, genetic diversity, DNA markers, ITS, *rbcL*, *cox3*, *cox1*

## Abstract

*Sargassum hemiphyllum* var. *chinense* is a common brown seaweed along the southeastern coast of China, playing a significant ecological role and possessing considerable resource utilization value. However, its genetic diversity and phylogeographic patterns remain poorly understood. In this study, we employed multiple molecular markers, including the nuclear ITS sequence (ribosomal internal transcribed spacer), the plastid *rbcL* gene (encoding the large subunit of ribulose-1,5-bisphosphate carboxylase/oxygenase), and the mitochondrial *cox3* and *cox1* genes (encoding cytochrome c oxidase subunits III and I, respectively), to elucidate the genetic and phylogeographic structure of *S. hemiphyllum* var. *chinense*. Our findings demonstrate that the combined use of plastid and mitochondrial gene sequences is suitable for phylogeographic studies of this species. Genetic structure difference was observed among 15 populations which localities covering most of its distribution range, likely resulting from colonization by ancestors of different origins and limited gene flow among populations. The study revealed two distinct lineages of *S. hemiphyllum* var. *chinense*, exhibiting a north–south geographical distribution with a mixed zone in the southern Fujian–eastern Guangdong coastal region. These lineages are inferred to have diverged during the Middle to Late Pleistocene due to the isolation of the East China Sea and South China Sea during glacial periods. Sub-lineage differentiation was also detected within the northern lineage. The southern lineage experienced demographic expansion following the end of the Last Glacial Maximum, while the northern lineage remained stable. The southern Fujian–eastern Guangdong region, characterized by high genetic diversity, may have served as a glacial refugium or a contact zone for the post-glacial recolonization of the two lineages. Global warming may lead to range contraction and reduced genetic diversity in this species. The high genetic diversity area should be prioritized for conservation efforts. Overall, these findings provide insights into the genetic structure status and causes of *S. hemiphyllum* var. *chinense* and offer a scientific basis for proposing reasonable measures for its resource management.

## 1. Introduction

*Sargassum*, a common brown algae genus widely distributed across temperate and tropical seas, is the largest genus within the class Phaeophyceae, comprising over 350 species [1]. *Sargassum* species hold significant economic value, serving as raw materials for extracting valuable bioactive compounds, feed for aquaculture, and food for human consumption [2,3,4,5]. Ecologically, they play a crucial role as foundational species in the construction of seaweed beds, forming underwater forests in coastal areas that enhance the local carbon sequestration capacity and provide food sources and habitats for various marine organisms [6,7,8,9]. *Sargassum hemiphyllum* is broadly distributed along the coastlines of the Northwest Pacific and is a commonly found seaweed growing on rocks in the lower intertidal zone. Taxonomically, it belongs to the subgenus *Bactrophycus* and section *Teretia*, characterized by linear and cuneiform leaves with truncate apices and a holdfast composed of branched cylindrical rhizoids [10,11,12]. *S. hemiphyllum* comprises two distinct varieties: *S. hemiphyllum* var. *chinense* and *S. hemiphyllum* var. *hemiphyllum*. Initially, these varieties were distinguished based on relatively significant differences in leaf morphology. Specifically, *S. hemiphyllum* var. *chinense* exhibits larger leaves, with basal primary leaves measuring 42–54 mm, whereas *S. hemiphyllum* var. *hemiphyllum* has smaller leaves, with basal primary leaves measuring 15 mm [10]. Subsequently, further molecular data have corroborated the validity of the division into two varieties, with multiple molecular markers from both nuclear and organellar genomes demonstrating differentiation between the two [13,14]. The distribution ranges of the two varieties do not overlap: *S. hemiphyllum* var. *chinense* is found along the coastlines from southeastern China to northern Vietnam, while *S. hemiphyllum* var. *hemiphyllum* is distributed along the coastlines of the Japanese archipelago and the eastern and southern coasts of the Korean Peninsula [13].

*S. hemiphyllum* var. *chinense* is one of the dominant species in subtropical warm water seaweed beds along the Chinese coast, forming a major component of several typical seaweed beds in the coastal provinces of Fujian, Guangdong, and Guangxi [15]. It plays a vital role in maintaining stable nearshore marine ecosystems in these regions. Previous research on this species has primarily focused on ecological applications, environmental adaptations, artificial cultivation, bioactive compound utilization, and comparative genomics [12,16,17,18,19,20]. Genetic diversity and population structure are crucial for developing resource utilization and conservation strategies, as these provide essential background information about the viability of populations and species. Investigations of more geographical populations across the distribution range would yield more robust data for understanding the species’ overall diversity and structure patterns. Phylogeographic studies can effectively reveal such patterns, thus proving critical for both species utilization and conservation. However, studies on the phylogeography of *S. hemiphyllum* var. *chinense* remain limited.

Phylogeography is a discipline that studies the spatial distribution of genetic lineages within and among closely related species. Substantial information about the demographic and historical nature of intraspecific evolution has been revealed through comparative phylogeographic assessments of many species [21]. Phylogeographic studies have been reported for multiple *Sargassum* species, covering geographic regions that include the Indian Ocean and the Pacific Ocean. These include species from the northwestern Pacific belonging to the subgenus *Bactrophycus*, such as *S. thunbergii* [22,23], *S. muticum* [24], *S. fusiforme* [25,26,27], and *S. horneri* [28,29,30,31], as well as species from the Indo-Pacific region belonging to the subgenus *Sargassum*, including *S. aquifolium* [32], *S. ilicifolium* [33], *S. polycystum* [34,35], and *S. plagiophyllum* [36]. Phylogeographic research on *S. hemiphyllum* var. *chinense* has only been mentioned in the studies of Cheang et al. [13,14]. In these studies, populations from only four locations were sampled, and analyses based on chloroplast *Rbc* spacer PCR-RFLP and sequence data, nuclear ITS2 sequences, and mitochondrial *Trn* spacer sequences revealed low genetic diversity. Specifically, only one haplotype was detected for chloroplast and nuclear markers, while mitochondrial markers exhibited just two haplotypes, suggesting an artefact of limited sampling or the use of inappropriate molecular markers. These previous studies were unable to capture the fine-scale population genetic structure and phylogeographic pattern of *S. hemiphyllum* var. *chinense*.

In this study, we collected specimens of the *S. hemiphyllum* var. *chinense* from 15 locations in the East China Sea and the South China Sea. These sampling sites cover a significant portion of the species’ distribution range. We used the sequences of nuclear internal transcribed spacer (ITS), chloroplast ribulose-1,5-bisphosphate carboxylase/oxygenase large subunit (*rbcL*) gene, and mitochondrial cytochrome c oxidase subunit III (*cox3*) and subunit I (*cox1*) genes to unravel the genetic diversity and phylogeographic patterns of this species. We also inferred the historical and contemporary factors that have shaped the current genetic structure. Our findings elucidate a more detailed phylogeographic pattern of *S. hemiphyllum* var. *chinense* and provide a scientific basis for the formulation of conservation strategies for this species.

## 2. Results

### 2.1. Genetic Diversity and Phylogenetic Analysis

ITS regions were amplified and sequenced from 300 individuals (20 individuals per population), among which 64 individuals exhibited intra-individual variation, containing multiple distinct ITS sequences. The sequenced ITS region had a length of approximately 1800 bp. After alignment and trimming, the sequence length was 1642 bp (including alignment gaps), with the actual sequence length ranging from 1628 to 1637 bp. This included the full-length regions of ITS1, 5.8S, and ITS2, with lengths of 878–887 bp, 159 bp, and 591 bp, respectively. The aligned sequences contained 14 insertion–deletion (indel) sites, all located in the ITS1 region, and 8 polymorphic sites, with 5 in ITS1, 1 in 5.8S, and 2 in ITS2. The average nucleotide compositions of T, C, A, and G in the ITS region were 22.6%, 26.9%, 16.8%, and 33.7%, respectively, with a C + G content of 60.6%. The C + G contents of ITS1, 5.8S, and ITS2 regions were 61.2%, 54.8%, and 61.5%, respectively.

A total of 13 ribotypes were identified from the ITS sequences. I-H1 was the most widely distributed, occurring in all populations and present in 280 individuals. I-H2 was found in four populations (Nanji (NJ), Zhangpu (ZP), Dingpeng (DP), and Mulun Bay (ML)), appearing in 36 individuals. I-H3 and I-H11 were present in three (Zhangpu (ZP), Dingpeng (DP), and Shanwei (SW)) and two (Wailingding (WL) and Naozhou (NZ)) populations, respectively. The remaining nine ribotypes were unique to single populations, with I-H12 and I-H6 being the most common, appearing in 20 and 11 individuals, respectively, and being endemic to the Xuwen (XW) and Keniaowei (KN) populations. A total of 11 ribotype combinations were identified within individuals, all consisting of two ribotypes. The most widely distributed combination was I-H1/I-H2, found in four populations (Nanji (NJ), Zhangpu (ZP), Dingpeng (DP), and Mulun Bay (ML)) and present in 35 individuals. This was followed by I-H1/I-H3 and I-H1/I-H11, each found in two populations (Zhangpu (ZP) and Dingpeng (DP), and Wailingding (WL) and Naozhou (NZ)), with four and two individuals, respectively. The remaining eight ribotype combinations were unique to single populations, with I-H1/I-H6 being the most common, appearing in 11 individuals from the Keniaowei (KN) population. Among the populations, the Shanwei (SW) population had the highest number of ribotypes (six), followed by Zhangpu (ZP) and Dingpeng (DP), each with four ribotypes. Five populations possessed endemic ribotypes, with Shanwei (SW) having four, Xuwen (XW) having two, and Zhangpu (ZP), Dingpeng (DP), and Keniaowei (KN) each having one endemic ribotype (Table S1, Supplementary materials).

Model selection based on the BIC indicated that the F81 model [37] was the best nucleotide substitution model for the ITS sequences. ML and BI phylogenetic trees constructed from the 13 ITS ribotypes showed that, except for I-H3 and I-H4 forming a stable clade, the other ribotypes did not form reliable clades. The terminal branches of the phylogenetic tree were short, indicating high similarity among ribotypes and no significant differentiation (Figure S1, Supplementary materials).

**Figure 1:**
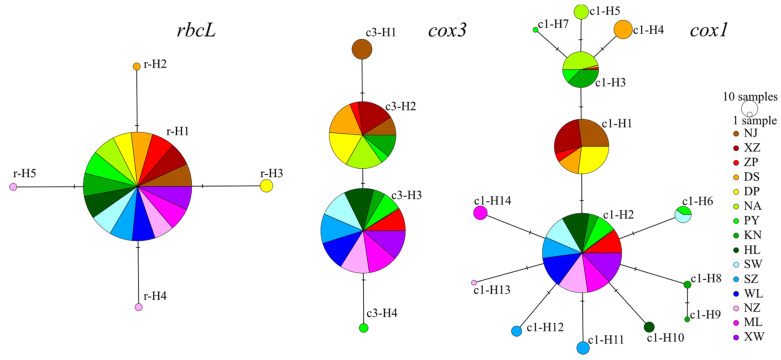
Median-joining network based on *rbcL*, *cox3* and *cox1* haplotypes. The size of circle is proportional to haplotype frequency. The one short bar on the connecting line represents one mutation step.

A total of 450 *rbcL* gene sequences were sequenced, with a length of approximately 1800 bp. After alignment and trimming, the full-length *rbcL* gene sequence of 1467 bp was obtained. The average nucleotide compositions of T, C, A, and G were 31.8%, 15.7%, 29.4%, and 23.0%, respectively, with a C + G content of 38.7%. There were four polymorphic sites, all of which were parsimony informative, and five haplotypes were identified. The overall haplotype diversity index was 0.053 ± 0.015, and the nucleotide diversity index was 0.00004 ± 0.00001 (Table S2). Among the five haplotypes, r-H1 was the most widely distributed, occurring in all populations with a total of 438 individuals. The other four haplotypes were endemic to single populations: r-H2 and r-H3 were found in the Dongshan (DS) and Dingpeng (DP) populations, with two and six individuals, respectively, while r-H4 and r-H5 were present in the Naozhou (NZ) population, each with two individuals. Among the 15 populations, 12 populations contained only one haplotype (r-H1). Among the remaining three populations, the Dingpeng (DP) population contained two haplotypes (r-H1 and r-H3) and exhibited the highest haplotype diversity and nucleotide diversity (0.331 ± 0.089 and 0.00023 ± 0.00006, respectively). The Naozhou (NZ) population had the highest number of haplotypes, with three (r-H1, r-H4, and r-H5). The Dongshan (DS) population contains two haplotypes (r-H1 and r-H2), with haplotype diversity and nucleotide diversity indices of 0.129 ± 0.079 and 0.00009 ± 0.00005, respectively (Table S2, Figure S2). The median-joining network of *rbcL* haplotypes is shown in Figure 1, where the five haplotypes formed a star-like structure, with r-H1 located at the center as the ancestral haplotype. The four surrounding haplotypes each differed from r-H1 by a single mutational step. The network did not exhibit a clear phylogeographic structure.

A total of 450 *cox3* gene sequences were sequenced, with a length of approximately 760 bp. After alignment and trimming, a partial *cox3* gene sequence of 741 bp was obtained, representing 90.48% of the full-length gene sequence (741 bp/819 bp). The average nucleotide compositions of T, C, A, and G were 39.2%, 16.7%, 20.2%, and 23.9%, respectively, with a C + G content of 40.6%. There were three polymorphic sites, all of which were parsimony informative, and four haplotypes were identified. The overall haplotype diversity index was 0.514 ± 0.013, and the nucleotide diversity index was 0.00076 ± 0.00003 (Table S3). Among the four haplotypes, haplotype c3-H3 was the most widely distributed, occurring in 11 populations ranging from southern Fujian to western Guangdong (from Zhangpu (ZP) to Xuwen (XW)), with a total of 266 individuals. This was followed by c3-H2, which was distributed across eight populations from Zhejiang to eastern Guangdong (from Nanji (NJ) to Keniaowei (KN)), with a total of 166 individuals. Haplotypes c3-H1 and c3-H4 were endemic, found in the Nanji (NJ) and Pingyu (PY) populations, with 15 and 3 individuals, respectively (Table S3, Figure S2). Among the 15 populations, 10 populations contained only one haplotype. The remaining five populations included Pingyu (PY), which contained three haplotypes, and Nanji (NJ), Zhangpu (ZP), Dongshan (DS), and Keniaowei (KN), each containing two haplotypes. The Nanji (NJ) and Pingyu (PY) populations had haplotype diversity indices greater than 0.5 (*H*_d_ = 0.517 ± 0.024, *H*_d_ = 0.508 ± 0.085, respectively), while the Pingyu (PY) population had the highest nucleotide diversity (*π* = 0.00075 ± 0.00015) (Table S3). The median-joining network of the *cox3* haplotypes is shown in Figure 1, where the four haplotypes formed a linear structure, with adjacent haplotypes differing by a single mutational step. The network of *cox3* haplotypes did not exhibit a clear phylogeographic structure.

A total of 450 *cox1* gene sequences were sequenced, with a length of approximately 1700 bp. After alignment and trimming, the full-length *cox1* gene sequence of 1587 bp was obtained. The average nucleotide compositions of T, C, A, and G were 39.8%, 16.8%, 20.9%, and 22.4%, respectively, with a C + G content of 39.2%. There were 13 polymorphic sites, including 10 parsimony informative sites, and 14 haplotypes were identified. The overall haplotype diversity index was 0.657 ± 0.018, and the nucleotide diversity index was 0.00063 ± 0.00003 (Table S4). The haplotype c1-H2 was the most widely distributed, occurring in 11 populations except for Nanji (NJ), Xiaozuo (XZ), Dingpeng (DP), and Nan’ao (NA), with a total of 234 individuals. Haplotype c1-H1 was found in five populations from Nanji (NJ) to Dingpeng (DP), with a total of 111 individuals. Haplotype c1-H3 was present in five geographically close populations in southern Fujian and eastern Guangdong, including Zhangpu (ZP), Dongshan (DS), Nan’ao (NA), Pingyu (PY), and Keniaowei (KN), with a total of 48 individuals. Haplotype c1-H6 was found in two populations, Pingyu (PY) and Shanwei (SW), with 10 individuals. The remaining 10 haplotypes were endemic to single populations (Table S5, Figure S2). Among the 15 populations, five populations—Nanji (NJ), Xiaozuo (XZ), Dingpeng (DP), Wailingding (WL), and Xuwen (XW)—each contained only one haplotype, with both haplotype diversity and nucleotide diversity indices being 0. Among the 10 populations containing multiple haplotypes, the Dongshan (DS) population had the highest haplotype diversity (*H*_d_ = 0.579 ± 0.047), while the Keniaowei (KN) population had the highest nucleotide diversity (*π* = 0.00079 ± 0.00011). The Naozhou (NZ) population had the lowest haplotype diversity and nucleotide diversity (*H*_d_ = 0.067 ± 0.061, *π* = 0.00004 ± 0.00004) (Table S4).

The best nucleotide substitution model, the HKY model [38], was used to construct ML and BI phylogenetic trees based on *cox1* haplotype sequences (Figure S3). The 14 haplotypes were divided into three clades in the phylogenetic tree: one clade included haplotypes c1-H3, c1-H4, c1-H5, and c1-H7; c1-H1 formed a separate clade; and the remaining nine haplotypes grouped into another clade. The median-joining network of *cox1* haplotypes (Figure 1) exhibited a structure consistent with the phylogenetic tree, displaying two star-like structures centered around c1-H3 and c1-H2, respectively. In the network, c1-H3 and c1-H2 were connected via c1-H1. Haplotypes that clustered with c1-H3 in the phylogenetic tree were distributed around c1-H3 in the network, while the eight haplotypes that grouped with c1-H2 in the phylogenetic tree were distributed around c1-H2 in the network. Each adjacent haplotype in the network differed by a single mutational step. Among the three clades identified in both the phylogenetic tree and the haplotype network, the c1-H1 clade was distributed across five populations ranging from Nanji (NJ) to Dingpeng (DP). The clade centered around c1-H3 was found in populations from the southern Fujian–eastern Guangdong region (Zhangpu (ZP), Dongshan (DS), Nan’ao (NA), Pingyu (PY), and Keniaowei (KN)). The clade centered around c1-H2 was distributed in 11 populations south of Zhangpu (ZP), excluding Dingpeng (DP) and Nan’ao (NA). Within the populations of Zhangpu (ZP), Dongshan (DS), Pingyu (PY), and Keniaowei (KN), haplotypes belonging to different clades were observed (Table S5, Figures S2 and S3).

**Figure 2:**
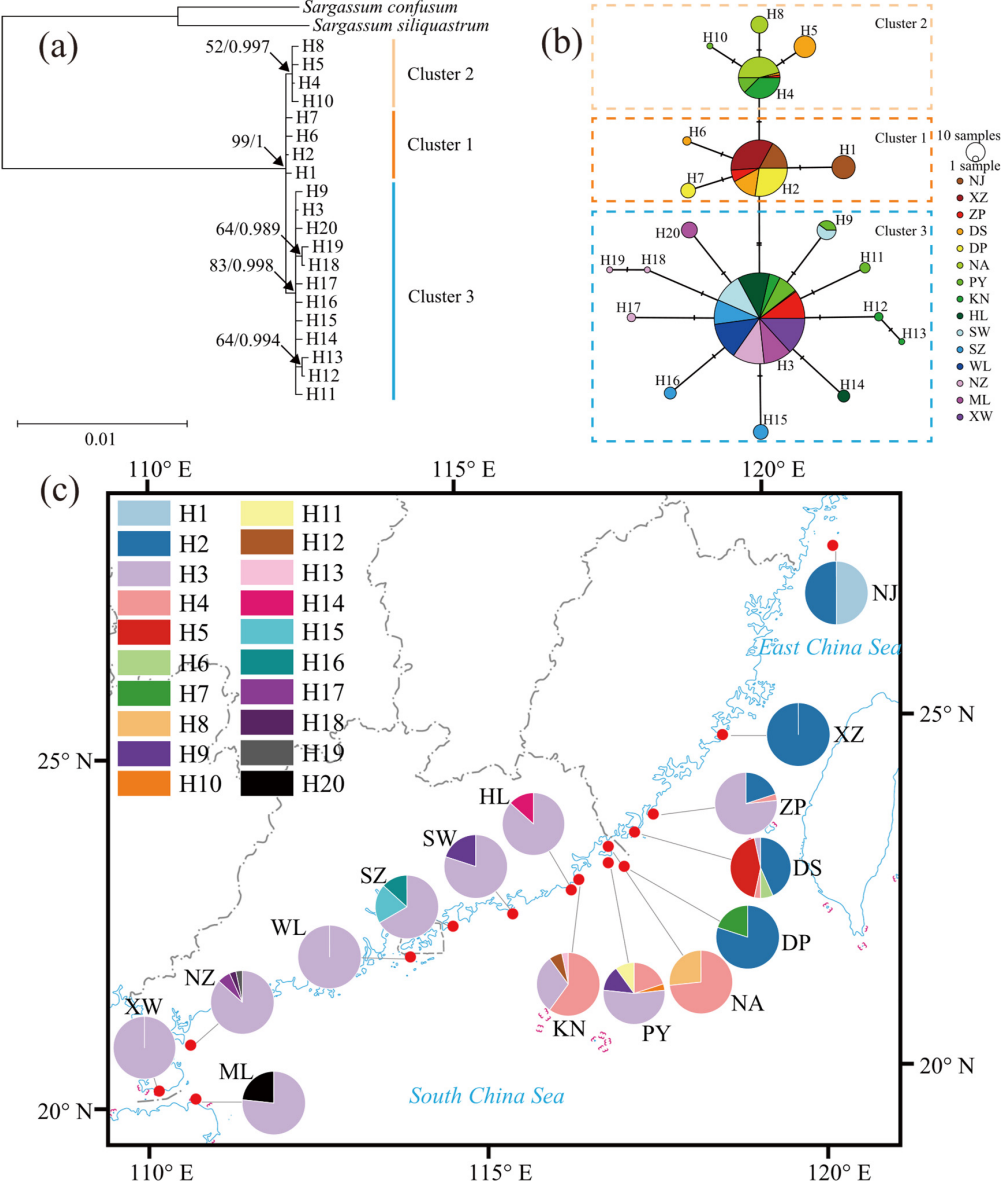
(**a**) Phylogenetic tree for concatenated sequence haplotypes. The 50% majority rule consensus tree. The nodes for which the bootstrap supports are greater than 50 for ML tree and have posterior probabilities greater than 0.9 for BI tree are labeled. (**b**) Median-joining network is based on concatenated sequence haplotypes. The size of circle is proportional to haplotype frequency. The one short bar on the connecting line represents one mutation step. (**c**) Geographical distribution and frequency of concatenated sequence haplotypes. Pie chart denotes the proportion of haplotypes present in each population.

The *cox3*, *cox1*, and *rbcL* sequences of each individual were concatenated to form a *cox3*–*cox1*–*rbcL* sequence, resulting in a total of 450 sequences with a length of 3795 bp. The average nucleotide composition was 36.6% T, 16.4% C, 24.1% A, and 22.9% G, with a C + G content of 39.3%. A total of 20 polymorphic sites were identified, including 3 singleton variable sites and 17 parsimony informative sites. Twenty haplotypes were recognized, with an overall haplotype diversity (*H*_d_) of 0.692 ± 0.020 and a nucleotide diversity (*π*) of 0.00042 ± 0.00001 (Table 1). Haplotype H3 was the most widely distributed, occurring in 11 populations except for Nanji (NJ), Xiaozuo (XZ), Dingpeng (DP), and Nan’ao (NA), and was represented by the largest number of individuals (228). Haplotype H2 was found in five populations from Nanji (NJ) to Dingpeng (DP), with 88 individuals. Haplotype H4 was present in five populations in the southern Fujian–eastern Guangdong region, including Zhangpu (ZP), Dongshan (DS), Nan’ao (NA), Pingyu (PY), and Keniaowei (KN), with 48 individuals. Haplotype H9 was observed in the Pingyu (PY) and Shanwei (SW) populations, with 10 individuals. The remaining 16 haplotypes were endemic, each occurring in only a single population (Figure 2c, Table S6). Among the 15 populations, Xiaozuo (XZ), Wailingding (WL), and Xuwen (XW) each contained only one haplotype, with both haplotype diversity and nucleotide diversity indices equal to 0. The other 12 populations contained 2 to 5 haplotypes each, with haplotype diversity indices ranging from 0.239 to 0.669 and nucleotide diversity indices ranging from 0.00006 to 0.00046 (Table 1). Among the populations, Dongshan (DS) and Pingyu (PY) had the highest number of haplotypes, with 5 each. The Pingyu (PY) population exhibited the highest haplotype diversity (0.669 ± 0.074), followed by the Nanji (NJ), Dongshan (DS), Keniaowei (KN), and Shenzhen (SZ) populations, all with haplotype diversity indices greater than 0.5. The Keniaowei (KN) population had the highest nucleotide diversity (0.00046 ± 0.00006), followed by the Dongshan (DS) and Pingyu (PY) populations, both with nucleotide diversity indices greater than 0.0003 (Table 1 and Table S6). The genetic diversity of the populations showed a trend of initially increasing and then decreasing from north to south.

Using the best nucleotide substitution model, the HKY model, ML and BI phylogenetic trees were constructed based on the concatenated *cox3*–*cox1*–*rbcL* sequences. The 20 haplotypes were divided into three clusters in the phylogenetic tree (Figure 2a). Specifically, Cluster 1 consisted of haplotypes H1, H2, H6, and H7; Cluster 2 included haplotypes H4, H5, H8, and H10; and Cluster 3 comprised the 12 remaining haplotypes. The median-joining network (Figure 2b) revealed that the 20 haplotypes formed three star-like structures, with haplotypes H2, H4, and H3 at the centers, each radiating multiple derived haplotypes. Haplotypes H1, H6, and H7 were clustered around H2; haplotypes H5, H8, and H10 were clustered around H4; and the remaining 11 haplotypes were clustered around H3. The distance between H2 and H3 was two mutational steps, while all other adjacent haplotypes were separated by a single mutational step. The structure of the haplotype network corresponded to the phylogenetic tree, with the star-like structures in the network forming the three clusters. Cluster 2 was connected to Cluster 3 through Cluster 1. In the phylogenetic tree and haplotype network, Cluster 1 was distributed across five populations, ranging from Nanji (NJ) to Dingpeng (DP); Cluster 2 was found in five populations in the southern Fujian–eastern Guangdong region, including Zhangpu (ZP), Dongshan (DS), Nan’ao (NA), Pingyu (PY), and Keniaowei (KN); and Cluster 3 was distributed in 11 populations south of Zhangpu (ZP), excluding Dingpeng (DP) and Nan’ao (NA). The Zhangpu (ZP) and Dongshan (DS) populations contained haplotypes belonging to all three clusters, while the Pingyu (PY) and Keniaowei (KN) populations contained haplotypes from Cluster 2 and Cluster 3. The 11 remaining populations each contained haplotypes exclusively from a single cluster (Figure 2b,c).

### 2.2. Population Genetic Structure

**Figure 3:**
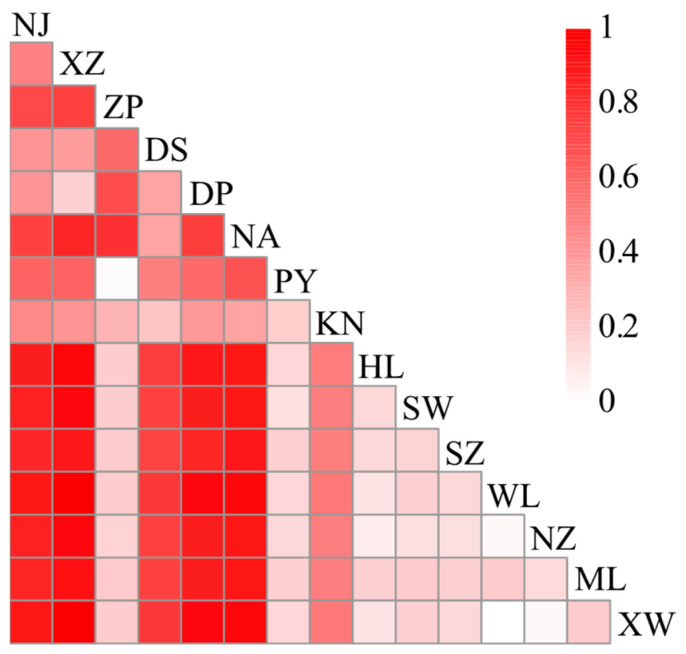
Heatmap of pairwise *F*_ST_ values based on *cox3*–*cox1*–*rbcL* concatenated sequences.

**Figure 4:**
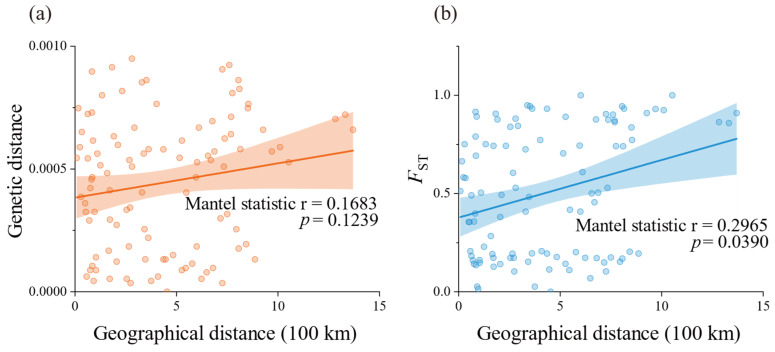
Mantel test of pairwise genetic distance and pairwise *F*_ST_ values with geographical distance among populations, respectively. The shadow of linear regression represents the 95% confidence interval.

Based on the concatenated *cox3*–*cox1*–*rbcL* sequence data, the pairwise genetic distances between populations ranged from 0 to 9.499 × 10⁻^4^ (Table S7), with relatively small genetic distances observed among all populations. Specifically, the genetic distance between the Wailingding (WL) and Xuwen (XW) populations was 0, indicating identical genetic structures between these two populations. The largest genetic distance (9.499 × 10⁻^4^) was found between the Nan’ao (NA) and Shenzhen (SZ) populations. Additionally, relatively large pairwise genetic distances were observed between Nan’ao (NA) and Mulang Bay (ML), Nan’ao (NA), and Shanwei (SW), and Nan’ao (NA) and Naozhou (NZ), all exceeding 0.0009. The *F*_ST_ values, used to measure the degree of genetic differentiation between populations, are shown in Figure 3 and Table S8. The maximum *F*_ST_ value of 1 was observed between the Xiaozuo (XZ) and Wailingding (WL) populations and between the Xiaozuo (XZ) and Xuwen (XW) populations, while the *F*_ST_ value between Wailingding (WL) and Xuwen (XW) was 0. Overall, the majority of population pairs exhibited large *F*_ST_ values with statistically significant differences (*p* < 0.05). Specifically, 23 population pairs had *F*_ST_ values between 0.15 and 0.25, and 66 pairs had *F*_ST_ values greater than 0.25, collectively accounting for 84.8% (89/105) of all population pairs (Table S8), indicating a substantial genetic structure differences among most populations. The Mantel test results (Figure 4) revealed no significant correlation between pairwise genetic distances and geographical distances (*p* = 0.1239, Table S9), and a statistically significant but weak correlation between pairwise *F*_ST_ values and geographic distance (*p* = 0.0390, r = 0.2965). These suggest that geographical isolation plays a limited role in shaping genetic differentiation among populations. AMOVA based on the concatenated *cox3*–*cox1*–*rbcL* sequence data from the 15 populations (Table 2) demonstrated significant genetic structure differences among populations, with the interpopulation genetic variation accounting for 66.25% of the total variation (*Φ*_ST_ = 0.66252, *p* < 0.001). Similar results were obtained from AMOVA of *cox3* and *cox1* sequence data (*Φ*_ST_ = 0.77293, *p* < 0.001; *Φ*_ST_ = 0.62867, *p* < 0.001). In contrast, the AMOVA of *rbcL* sequence data revealed that genetic variation primarily occurred within populations, with a lower proportion of interpopulation genetic variation (*Φ*_ST_ = 0.10345, *p* < 0.001), which is attributed to the fact that most populations shared a single common *rbcL* haplotype.

**Figure 5:**
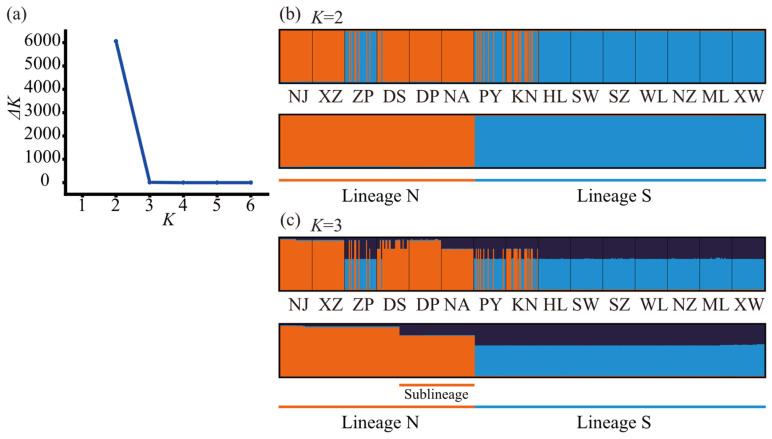
(**a**) Distribution of Δ*K* in populations’ genetic structure analysis based on *cox3*–*cox1*–*rbcL* concatenated sequences. Q value assignments of individuals under (**b**) *K* = 2 and (**c**) *K* = 3, organized by sampling populations and lineages.

**Figure 6:**
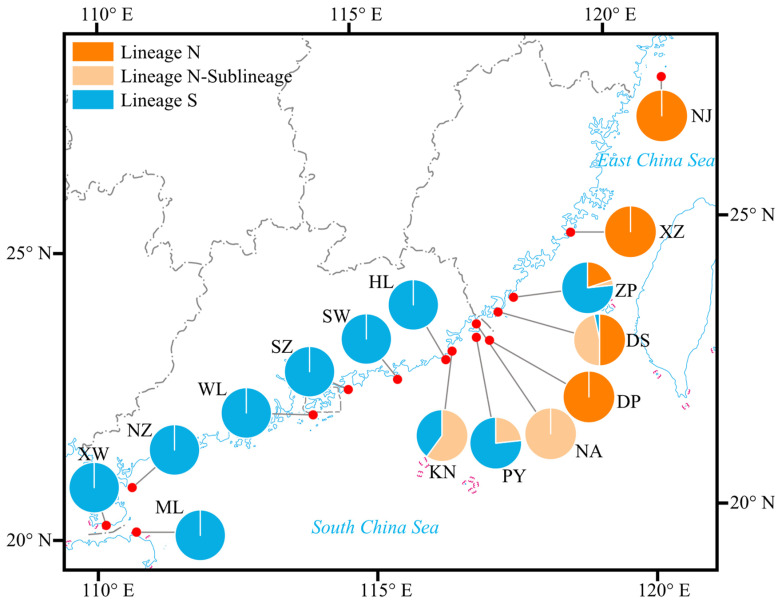
Geographical distribution and frequency of lineages. Pie chart denotes the proportion of lineages present in each population.

Using the *cox3*–*cox1*–*rbcL* concatenated sequence data from all individuals, a structure analysis of genetic lineage structure was conducted. The results indicated that the optimal number of genetic clusters was 2, suggesting the presence of two hypothetical ancestral populations, as the Δ*K* value reached its maximum at *K* = 2 (Figure 5a). The genetic assignments of individuals under *K* = 2, represented by two colors, is shown in Figure 5b. Based on the composition of genetic assignments, all individuals could be classified into two lineages, which exhibited a north–south geographical distribution (Figure 6). These lineages were labeled as Lineage N and Lineage S, respectively. The two lineages showed distinct differences, with predominantly pure genetic origins and only a minor admixture from their hypothetical ancestral sources (Figure 5b). Among the 15 populations, all individuals from the relatively northern populations—Nanji (NJ), Xiaozuo (XZ), Dingpeng (DP), and Nan’ao (NA)—were assigned to Lineage N. In contrast, all individuals from the relatively southern populations—Huilai (HL), Shanwei (SW), Shenzhen (SZ), Wailingding (WL), Naozhou (NZ), Mulang Bay (ML), and Xuwen (XW)—were assigned to Lineage S. The four populations located in the southern Fujian-eastern Guangdong region—Zhangpu (ZP), Dongshan (DS), Pingyu (PY), and Keniaowei (KN)—exhibited an admixture of both lineages (Figure 5b and Figure 6). An AMOVA of the two lineages revealed that the genetic variation between lineages accounted for 79.22% of the total variation (*Φ*_ST_ = 0.79218, *p* < 0.001), indicating significant differentiation between the two lineages (Table 2).

### 2.3. Divergence Time and Historical Demographic Dynamics

**Figure 7:**
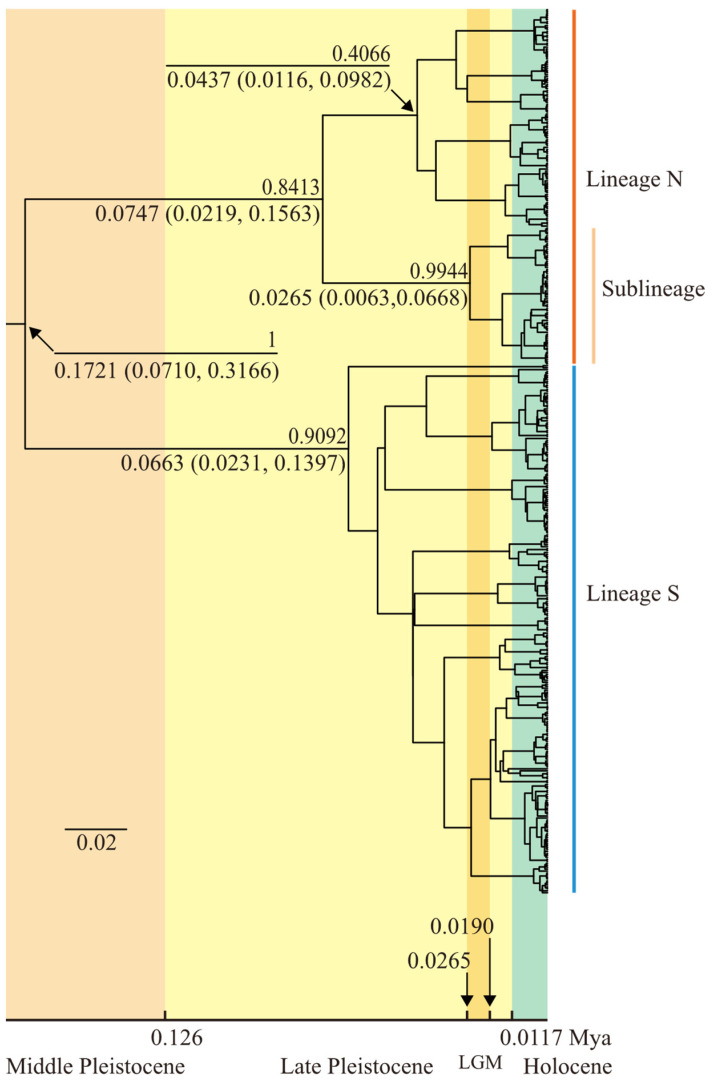
Calibrated gene tree of concatenated sequence. The numbers above nodes are Bayesian posterior probabilities. The numbers below nodes are nodes ages and 95% highest posterior density (95% HPD) confidence intervals. Different background colors represent distinct geological periods. The timeline at the bottom is scaled in million years ago (Mya).

Using the *cox3*, *cox1*, and *rbcL* sequence data from all 450 individuals, a calibrated gene tree was generated under the conditions of the average mutation rates for each sequence, the best nucleotide substitution models (*cox3*: HKY model; *cox1*: HKY model; *rbcL*: F81 model), and a Bayesian skyline tree prior model, as shown in Figure 7. The structure of the calibrated gene tree indicates that all individuals cluster into two clades with high support, with posterior probabilities of 84.1% and 90.9%, respectively. This suggests that all individuals can be divided into two lineages, which is consistent with the lineage division results from the aforementioned structure analysis. The divergence time between the two lineages is estimated to be 0.1721 Mya (million years ago) (95% HPD confidence interval: 0.0710–0.3166 Mya). The time to most recent common ancestor (TMRCA) for Lineage N is estimated to be 0.0747 Mya (95% HPD confidence interval: 0.0219–0.1563 Mya), while the TMRCA for Lineage S is estimated to be 0.0663 Mya (95% HPD confidence interval: 0.0231–0.1397 Mya). Within Lineage N, a highly supported subclade with a posterior probability of 99.44% indicates the presence of a sublineage differentiation. Similarly, in the structure analysis, when *K* = 3, the Q value assignment also revealed the existence of this sublineage (Figure 5c). The TMRCA for this sublineage is estimated to be 0.0265 Mya (95% HPD confidence interval: 0.0063–0.0668 Mya). In contrast, no highly supported subclades were formed within Lineage S, indicating a lack of significant genetic differentiation within this lineage.

**Figure 8:**
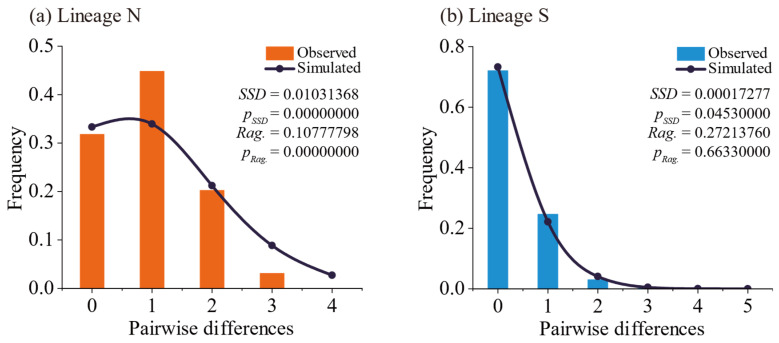
Mismatch distribution of two lineages based on *cox3*–*cox1*–*rbcL* concatenated sequence.

**Figure 9:**
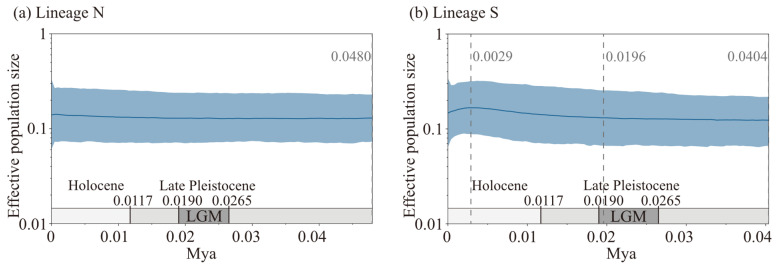
Bayesian skyline plots of two lineages. The upper and lower limits of blue trend represent the 95% HPD confidence intervals. The curves correspond to the median values. Vertical dashed lines with time labels mark historical demographic events. Gray bands represent geological periods.

Neutrality tests were conducted on the two lineages based on the *cox3*–*cox1*–*rbcL* concatenated sequence data. The results showed that Lineage N exhibited non-significant negative values for both Tajima’s *D* and Fu’s *F*_S_ (*p* > 0.1), while Lineage S displayed significantly negative values for both statistics (*p* < 0.001) (Table 3). The neutrality test results suggest that Lineage N did not significantly deviate from the neutral evolution model, indicating that it may not have undergone demographic expansion. In contrast, Lineage S significantly deviated from the neutral model, suggesting a possible historical demographic expansion. Mismatch distribution analyses for the two lineages were performed using the *cox3*–*cox1*–*rbcL* concatenated sequence data under the spatial expansion model. The observed and simulated values are presented in Figure 8. For Lineage N, although the observed values showed a unimodal distribution, they did not match the simulated curve, and both the *SSD* and *Rag.* statistics were significant *(p*_SSD_ = 0.0000, *p*_Rag._ = 0.0000) (Figure 8a), rejecting the hypothesis of demographic expansion. For Lineage S, the observed values closely matched the simulated curve, with a significant *SSD* statistic (*p*_SSD_ = 0.0453) but a non-significant *Rag.* statistic (*p*_Rag._ = 0.6633) (Figure 8b), providing insufficient evidence to reject the demographic expansion hypothesis. This indicates that Lineage S may have undergone expansion. Bayesian skyline reconstruction was performed using the *cox3*, *cox1*, and *rbcL* sequence data to infer the historical effective population size changes of the two lineages over time (Figure 9). For Lineage N, the effective population size remained nearly stable from approximately 48.0 kya (thousand years ago) onward, showing no significant trend of demographic expansion (Figure 9a). In contrast, Lineage S exhibited an initial expansion followed by a decline in effective population size starting around 40.4 kya. The demographic expansion began approximately 19.6 kya, followed by a demographic decline around 2.9 kya (Figure 9b). The Bayesian skyline reconstruction results are consistent with the conclusions drawn from the neutrality tests and mismatch distribution analyses, supporting the inferred historical demographic dynamics of the two lineages.

## 3. Discussion

### 3.1. Variability and Applicability of the Genetic Markers

The ITS sequence, as a non-protein-coding region of the nuclear genome, has been widely utilized in phylogeographic studies of various brown algae due to its appropriate evolutionary rate [23,34,36,39]. Furthermore, some studies have indicated that the ITS sequences in brown algae may exhibit incomplete concerted evolution, where multiple distinct ITS sequences exist within a single individual, often exceeding two, suggesting variations among multiple ITS copies within the nuclear genome rather than a simple heterozygous state [13,33]. Although the ITS sequences of all samples in this study showed no more than two ribotype combinations, previous research [13] has demonstrated intragenomic variations in the ITS2 of the model species *S. hemiphyllum*. Since this study could not determine the specific copy number of ITS sequences in the nuclear genome or the proportional relationships among different ITS sequences within individual, the frequencies of ribotypes within individual and population could not be ascertained. Consequently, this study only records the types of ITS ribotypes obtained, without a statistical analysis of their frequencies or their application in population genetic structure analysis. In prior studies, only one ITS2 ribotype was identified in *S. hemiphyllum* var. *chinense* samples from southern China [13], whereas this study identified 13 ribotypes, likely due to the inclusion of more populations and a longer sequencing region encompassing ITS1, 5.8S, and ITS2. The ribotype I-H1 was widely distributed, and its sequence matched the published sequence JF710313 on GenBank. Except for I-H5 and I-H6, the ITS2 regions of the other ITS ribotypes in this study were consistent with the ITS2 sequence (GenBank: FJ712722) of *S. hemiphyllum* var. *chinense* from southern China published on GenBank. The ribotypes I-H8 and I-H10 from the SW population contained one and two insertions, respectively, at the same sites as the ITS sequence (GenBank: AY150006) of *S. hemiphyllum* from Cheju Island, Korea [40], which may provide evidence of hybridization between *S. hemiphyllum* and *S. hemiphyllum* var. *chinense*. This aligns with the findings of Cheang et al. [13], who inferred the possibility of hybridization between the two based on the presence of identical haplotypes in *S. hemiphyllum* and *S. hemiphyllum* var. *chinense*. Current research indicates that the distribution ranges of *S. hemiphyllum* and *S. hemiphyllum* var. *chinense* do not overlap, with the freshwater input from the Yellow and Yangtze Rivers and the soft substrates formed by river sediments along the coast acting as barriers to their distribution [13]. Although *Sargassum* germlings have limited dispersal capabilities [41], studies have shown that floating thalli of *Sargassum* species can migrate over long distances via ocean currents, such as the South China Sea Warm Current, Kuroshio Current, and China Coastal Current [33,42]. Thus, different varieties of *S. hemiphyllum* may drift into each other’s distribution ranges and produce gametes, leading to hybridization. The diversity of ITS sequences in the nuclear genome provides evidence for hybridization between varieties.

Plastid and mitochondrial genes, due to their appropriate levels of variation among species or individuals within a species, have also become commonly used genetic markers for phylogeographic studies. For example, in green algae (e.g., *Ulva* [43]), the plastid gene *TufA* is frequently utilized. In red algae (e.g., *Agarophyton vermiculophyllum*, *Pterocladiella capillacea* [44,45]), genes such as *cox1* and *rbcL* are commonly employed. In brown algae (e.g., *S. horneri*, *S. plagiophyllum* [28,31,36]), *cox3*, *cox1*, and *rbcL* are typically used. In this study, the sequencing of genetic markers was conducted on 450 individuals from 15 geographic populations, with *rbcL*, *cox3*, and *cox1* identifying 5, 4, and 14 haplotypes, respectively. Among the three markers considered, *cox1* exhibited the highest variability and displayed a genetic structure correlated with geographic distribution, making it a suitable marker for investigating intraspecific variation in *S. hemiphyllum* var. *chinense*. In previous studies, the genetic markers *Rbc* and *TrnW-I* identified only one and two haplotypes, respectively, in 57 and 39 samples of this species from four geographic populations [13]. For other species within the *Sargassum* genus, such as *S. horneri*, *rbcL* and *cox3* are appropriate markers, with *rbcL* identifying 9 haplotypes from 144 sequences and *cox3* detecting 51 haplotypes from 361 specimens [28]. Similarly, *cox1* identified 16 haplotypes in 268 individuals of *S. ilicifolium* from 23 populations [33] and 9 haplotypes in 351 individuals of *S. plagiophyllum* from 10 populations [36]. This indicates that the degree of intraspecific variation in genetic markers varies among different species within the *Sargassum* genus, and the suitability of genetic markers for phylogeographic analysis differs between species within the genus.

*S. hemiphyllum* var. *chinense* is an oogamous brown alga, with mitochondria and plastids inherited maternally [46]. Therefore, the mitochondrial gene markers *cox3* and *cox1*, as well as the plastid gene marker *rbcL*, exhibit synchronized inheritance patterns. In this study, the combined analysis of mitochondrial and plastid genes provides a more comprehensive reflection of intraspecific variation in *S. hemiphyllum* var. *chinense* and yields additional phylogeographic insights. The concatenated sequences identified more haplotypes than individual marker sequences, identifying 20 haplotypes among 450 individuals, with overall higher haplotype diversity. Both the phylogenetic tree and haplotype network revealed distinct lineage structures. In this study, *rbcL* and *cox3* produced fewer haplotypes and did not exhibit a clear phylogenetic structure, making it difficult to accurately reflect the genetic lineage patterns of the species. However, when combined with *cox1*, another organelle gene, they could provide additional potential insights into maternal inheritance. Similar approaches have been applied in studies of other seaweeds, such as the combined use of *rbcL* and *cox1* genetic markers to investigate the differences and connections among the geographic populations of the red algae *Pterocladiella capillacea* [44].

### 3.2. Genetic Variability and Population Structure

Previous studies have indicated that the distribution range of *S. hemiphyllum* var. *chinense* extends from Zhoushan Archipelago in China to the coastal area of Vietnam [10,13,14,47]. In this study, the sampling range spanned from Nanji Island in the East China Sea to the Qiongzhou Strait in the South China Sea, covering a significant portion of the species’ distribution. Consequently, the findings of this study provide a comprehensive representation of the current genetic status of this species. Overall, *S. hemiphyllum* var. *chinense* exhibits high haplotype diversity and low nucleotide diversity, a pattern similar to other species within the genus, such as *S. fusiforme* [26] and *S. ilicifolium* [33], based on their respective mitochondrial gene data. When considering the number of haplotypes, haplotype diversity, and nucleotide diversity collectively, the genetic diversity of *S. hemiphyllum* var. *chinense* across the sampling range generally shows an initial increase followed by a decrease from north to south. Among the populations, DS, PY, and KN, which are geographically close to each other, exhibit relatively high haplotype diversity and nucleotide diversity, along with significant genetic structure differences (*F*_ST_: 0.18–0.49). In contrast, the three populations with the lowest genetic diversity each contain only a single haplotype, with WL and XW sharing the same haplotype. The variation in genetic diversity among populations may reflect differences in their environmental adaptability and extinction risks. Low genetic diversity could exacerbate declines in adaptability, leading to reduced reproduction and increased mortality [39].

The *F*_ST_ values revealed a substantial level of genetic differentiation among populations, and the AMOVA results further indicated pronounced genetic structure differences between these populations. In this study, the 20 haplotypes identified from the concatenated sequences exhibited distinct and consistent clustering topologies in both the phylogenetic tree and the haplotype network, with their distribution across different populations showing a geography-related pattern. Specifically, three shared haplotypes, which can be called ancestral haplotypes (H2, H3, H4), appeared in numerous individuals and displayed a location-dependent distribution, roughly aligning in a north-to-south arrangement. Populations sharing the same haplotype were geographically contiguous. All endemic haplotypes were found within the same geographic population as their ancestral haplotypes. However, the Mantel test based on concatenated sequence data showed no significant correlation between pairwise genetic distances and geographic distances among populations, with only a weak correlation observed between *F*_ST_ values and geographic distances (Figure 4). This suggests that the current genetic structure differences among populations are not due to isolation by distance but rather result from colonization by ancestors of different origins. These colonizing ancestors generated endemic haplotypes within their respective populations, leading to the observed genetic structure differences. Additionally, this pattern indicates limited gene flow among the populations.

### 3.3. Phylogeographic Structure and Demographic History

Previous studies have indicated that the divergence time between *S. hemiphyllum* and *S. hemiphyllum* var. *chinense* occurred between 6.58 and 11.25 Mya [13], during the late Miocene. In contrast, this study estimated the divergence time of the two lineages of *S. hemiphyllum* var. *chinense* to be 0.1721 Mya (95% HPD: 0.0710–0.3166 Mya), during the mid-Pleistocene to late Pleistocene, which is significantly later and represents a reasonable inference. The Sea of Japan basin, formed during the middle Miocene, served as a refugium for *S. hemiphyllum*, while the ancestor of *S. hemiphyllum* var. *chinense* originated from a refugium located in the South China Sea [13]. This refugium was formed by the land bridge connecting mainland China and Taiwan Island, which isolated the South China Sea from the East China Sea [48,49]. The two lineages of *S. hemiphyllum* var. *chinense* exhibit a north–south geographical distribution, and their divergence may have been driven by the geographical separation of the East China Sea and South China Sea during the Pleistocene glaciation. It is hypothesized that *S. hemiphyllum* var. *chinense*, originating from the South China Sea refugium, expanded into the East China Sea during interglacial periods and subsequently became isolated during the Pleistocene glaciation, leading to the formation of the two lineages. The glacial refugium of Lineage N may have been located in the Okinawa Trough basin. During glacial periods, when sea levels dropped, the Yellow Sea, Bohai Sea, and East China Sea retreated, exposing the continental shelf, while the marginal sea retreated into the Okinawa Trough, which was bounded by the East China Sea continental shelf and the Ryukyu Islands [48,50]. The Okinawa Trough, stretching from northeast Taiwan Island to Kyushu Island, is geographically close to the current distribution range of Lineage N in its southwestern part. The hypothesis of the Okinawa Trough as a glacial refugium has also been supported by studies on *S. ilicifolium* [33]. The sublineage within Lineage N is distributed in the southern Fujian-eastern Guangdong coastal region, with the TMRCA estimated at 0.0265 Mya, during the last glacial maximum (LGM) of the late Pleistocene. This sublineage likely formed due to internal isolation within Lineage N during the LGM.

The vicinity of the southern Fujian–eastern Guangdong coastal region may have served as a glacial refugium, as evidenced not only by the distribution of Lineage N sublineage in this region but also by the higher haplotype diversity and the presence of multiple endemic haplotypes, which are key signatures of glacial refugia [51,52,53]. This inference aligns with the hypothesis proposed by Hu and Duan [54] that a glacial refugium might have existed in the southwestern region of Taiwan Island, given the close proximity of the southern Fujian–eastern Guangdong area to southwestern Taiwan. However, due to the limited sampling sites in the East China Sea in this study, the speculation regarding the refugium may lack precision. Additional sampling from the northern and southern regions of Taiwan Island would provide more comprehensive insights into the origin of lineages and the potential locations of glacial refugia.

Although high genetic diversity is a characteristic of glacial refugium, it is not the sole criterion for identifying such refugium. The recolonization of different lineages from separate refugia in the same region can also create a high genetic diversity structure [54]. In this study, the southern Fujian–eastern Guangdong region exhibits high genetic diversity, multiple endemic haplotypes, and a mixed distribution of two lineages. Therefore, the current high genetic diversity in this region may also result from the recolonization of the two lineages following the end of glacial isolation. Based on the demographic history inference, Lineage S underwent population expansion at 0.0196 Mya, a time that coincides with the end of the LGM. This infers that, as sea levels rose at the end of the LGM, Lineage S expanded from glacial refugium and colonized new habitats. In the southern Fujian–eastern Guangdong coastal region, secondary contact occurred due to the northern and southern lineages recolonization, establishing a contact zone with high genetic diversity.

### 3.4. Insights of Species Conservation

Numerous studies have indicated that, under the context of climate change, seaweeds may shift to higher latitudes or greater depths in search of more suitable habitats [55]. *S. fusiforme* and *S. thunbergii*, two common brown algae, share overlapping distribution ranges and occupy similar ecological niches in the intertidal zone with *S. hemiphyllum* var. *chinense*. Research on these two species predicts that their suitable distribution areas will shift northward under future climate conditions, with a loss of distribution in middle-low latitude regions [56]. *S. hemiphyllum* var. *chinense* is distributed in subtropical regions, and rising sea surface temperature may lead to the decline in southern populations due to their inability to adapt to a higher temperature. Meanwhile, the northern distribution boundary of this species is constrained by the freshwater influx from the Yellow River and Yangtze River, as well as the muddy soft bottom along the coastal areas, which cannot provide the suitable salinity conditions for growth or rocky substrates for attachment [13,14]. This limits the potential for habitat expansion to the north. Consequently, global warming is likely to reduce the current distribution range of *S. hemiphyllum* var. *chinense* and lead to the loss of endemic haplotypes in southern populations, leading to a decline in the genetic diversity of this species.

High levels of genetic diversity are crucial for maintaining the evolutionary potential and survival capacity of species [39]. Under climate change conditions, high biodiversity can enhance the survival rates of macroalgae [57]. Therefore, preserving existing biodiversity is of significant importance for the conservation of *S. hemiphyllum* var. *chinense*. The results of this study reveal that *S. hemiphyllum* var. *chinense* exhibits a high proportion of endemic haplotypes (17/21) and majority populations possessing endemic haplotypes (9/15), along with high pairwise *F*_ST_ values among populations. These genetic structure characteristics indicate limited gene flow between different populations and poor dispersal ability. Consequently, to maintain the genetic diversity of *S. hemiphyllum* var. *chinense*, priority should be given to protecting existing habitats to sustain different geographical populations, with particular attention focused on the southern Fujian–eastern Guangdong coastal region, which exhibits high genetic diversity of this species and can be regarded as a significant gene pool. Conservation measures could be implemented through establishing marine protected areas. Within these protected areas, the harvesting of algal thalli should be strictly regulated or prohibited, and destructive human activities must be prevented, including coastal construction projects that degrade intertidal rocky substrates as well as pollution discharges that elevate water turbidity, increase sedimentation, or lead to eutrophication. Furthermore, regular monitoring of different regional populations should be conducted, including field surveys during the rapid growing stage and reproductive periods (February to May [19]) to track population dynamics. On the other hand, scientific and technological approaches should be adopted. This includes developing laboratory preservation and mariculture technologies for germplasm conservation, with emphasis on protecting the genetic resources of rare endemic haplotypes. Concurrently, seaweed bed restoration techniques should be investigated for natural population recovery.

## 4. Materials and Methods

### 4.1. Seaweed Collection and Molecular Procedures

**Figure 10:**
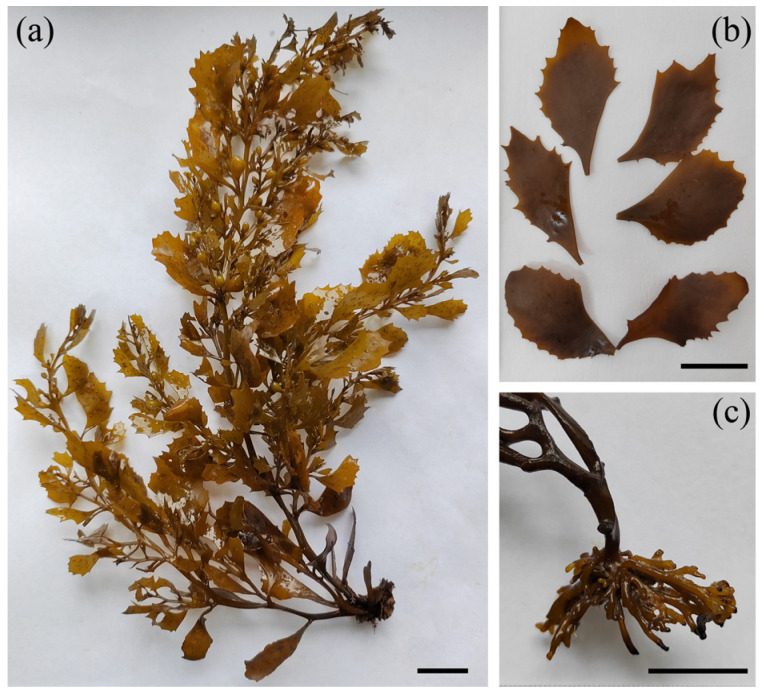
*Sargassum hemiphyllum* var. *chinense*: (**a**) Entire thallus; (**b**) leaves with truncated apex; and (**c**) holdfast composed of branched cylindrical rhizoids. Scale bars each represent 2 cm.

Based on historical records of *S. hemiphyllum* var. *chinense* distribution [47] and prior field experience, extensive sampling was conducted across China’s intertidal zones from January 2021 to February 2023 to maximize geographical coverage. Collections were performed during late winter to early summer, corresponding to the species’ rapid growth and reproductive phases. After surveying potential habitats, specimens were obtained from 15 sites (Table 1, Figure 2c), spanning four coastal provinces (Zhejiang, Fujian, Guangdong, Hainan), covering most of the species’ distribution range. To avoid sampling specimens from the same maternal organism, collected individuals were spaced at least 1 m apart at each site [13]. Whole thalli or apical branches with leaves (10–20 cm in length) were collected, rinsed with filtered seawater to remove attached epiphytes, small animals, and debris, and stored in plastic bags at −20 °C (the thallus and its main characteristics are shown in Figure 10). The genetic pattern of each population was represented by the collected samples. Although analyzing more samples per population would provide more information, it would require greater economic and time investment. Based on previous research methods [14] and considering the balance between cost and benefit, we randomly selected 30 individuals from each sampling site to constitute geographic populations, resulting in 15 populations totaling 450 individuals.

Approximately 0.1 g of leaf tissue from each individual was used for total DNA extraction. The Plant Genomic DNA Kit (DP305, TIANGEN, Beijing, China) was used according to the manufacturer’s instructions, and the extracted DNA was stored at −20 °C. Four molecular markers were selected for analysis: the nuclear internal transcribed spacer (ITS) region, the chloroplast gene encoding the large subunit of ribulose-1,5-bisphosphate carboxylase/oxygenase (*rbcL*), and the mitochondrial genes encoding cytochrome c oxidase subunit III (*cox3*) and subunit I (*cox1*). Four primer pairs were used for sequence amplification: LB1(F) (5′-CGCGAGTCATCAGCTCGCATT-3′) and LB2(R) (5′-AGCTTCACTCGCCGTACTGG-3′) [58] for the full-length ITS sequence; rbcL-S-by1 (5′-AAGGGGACTTATAATGTACTCCGTA-3′) (designed in this study) and rbcS-P1 (5′-GGATCATCTGYCCATTCTACAC-3′) [59] for the full-length *rbcL* gene; trnY-P1 (5′-TCYATCRTAGGTTCGAATCC-3′) and cox3-P2 (5′-ACAAARTGCCAATACCAAGC-3′) [60] for a partial *cox3* sequence; and 0909cox1F (5′-GTGGTTATTATTGGATGGACTG-3′) and 1010cox1R (5′-CTCTACCACTGAGTTATAGGC-3′) (designed in this study) for the full-length *cox1* gene. PCR amplification was performed using 2× Rapid Taq Master Mix (P222, Vazyme, Nanjing, China) in a 25 μL reaction volume. The PCR program consisted of an initial denaturation at 95 °C for 3 min, followed by 35 cycles of 95 °C for 15 s, annealing for 15 s, and 72 °C for 20 s, with a final extension at 72 °C for 5 min. The annealing temperatures were 60 °C for ITS, 48 °C for *cox3*, and 57 °C for *rbcL* and *cox1*. PCR products were sequenced by BGI Tech Solutions (Beijing Liuhe) Co., Ltd. (Beijing, China) using the amplification primers. For ITS sequences showing overlapping peaks, PCR products were gel-purified using the TIANgel Midi Purification Kit (DP209, TIANGEN, Beijing, China), followed by TA cloning and transformation using the pUCm-T Vector Rapid Cloning Kit (B522214, Sangon Biotech, Shanghai, China) and DH5α Competent Cells (B528413, Sangon Biotech, Shanghai, China). Positive clones were selected using blue-white screening, cultured in liquid medium, and sequenced.

Sequencing results were aligned, edited, and manually corrected using ChromasPro v2.1.3 and MEGA v11.0.11 [61], with reference to published sequences (GenBank: JQ807792, JF710313, AB043779, MT800998, KM210510). The amplified sequences were confirmed by BLASTN v2.16.1+ analysis on the NCBI website (https://www.ncbi.nlm.nih.gov (accessed on 30 August 2023)). Sequences from each individual were concatenated in the order of *cox3*, *cox1*, and *rbcL* to generate the *cox3*–*cox1*–*rbcL* concatenated sequence.

### 4.2. Genetic Diversity, Phylogeny, and Population Genetic Structure

Base composition, polymorphic sites, parsimony informative sites, nucleotide diversity (*π*), the number of haplotypes, and haplotype diversity (*H*_d_) were calculated using the software DnaSP v5.10.01 [62]. Prior to constructing phylogenetic trees with haplotype sequences, a test of substitution saturation was performed in DAMBE v7.3.32 [63] using the method of Xia et al. [64]. After confirming the absence of saturation, the best nucleotide substitution model was determined based on the Bayesian information criterion (BIC) in jModelTest v2.1.10 [65,66]. Maximum likelihood (ML) phylogenetic trees were constructed using MEGA v11.0.11, with outgroups selected as *S. thunbergii* (GenBank: JQ807793), *S. muticum* (GenBank: KY047242), *S. siliquastrum* (GenBank: ON675449, ON675443), and *S. confusum* (GenBank: NC_066460, NC_066050). Bootstrap replications were set to 1000. Bayesian inference (BI) phylogenetic trees were constructed using the MrBayes [67] plugin in PhyloSuite v1.2.3 [68,69], with the same outgroups. The Markov chain Monte Carlo (MCMC) process was run for 1.2 million generations, with a sampling frequency of 1000, 4 chains, and 2 independent runs. A burn-in fraction of 0.25 was applied. Convergence was confirmed when the average standard deviation of split frequencies fell below 0.01. A 50% majority rule consensus tree was generated from the results. The tree was visualized using FigTree v1.4.4, with posterior probabilities displayed at the nodes. Haplotype networks were constructed using the median-joining method [70] in PopART v1.7 [71].

Genetic distances between populations were calculated using the Tamura–Nei model [72] in MEGA v11.0.11, based on *cox3*–*cox1*–*rbcL* concatenated sequences from all individuals (the model was determined by jModelTest v2.1.10). Pairwise *F*_ST_ (Fixation Index) between populations were computed in Arlequin v3.5.2.2 [73], with statistical significance tested using 10,000 permutations. Geographic distances between the 15 populations were measured using the map distance tool on the National Platform for Common GeoSpatial Information Services (MAP WORLD) (https://www.tianditu.gov.cn (accessed on 27 April 2024)). Mantel tests were performed in R using the tidyverse and vegan packages to analyze correlations between genetic distance and geographic distance, as well as between the *F*_ST_ and geographic distance, with statistical significance assessed using 1000 permutations.

Population genetic structure analysis was conducted using Structure v2.3.4 [74] on *cox3*–*cox1*–*rbcL* concatenated sequences from all individuals. Parameters included a burn-in period of 200,000 steps and 1 million MCMC repetitions after burn-in. The admixture model and allele frequencies correlated model were selected. The number of clusters (*K*) was set from 1 to 7, with 10 iterations per *K*. Results were uploaded to StructureSelector (https://lmme.ac.cn/StructureSelector/ (accessed on 19 October 2024)) [75] to determine the optimal *K* value using the Evanno method [76] and to generate population structure plots [77].

Analysis of molecular variance (AMOVA) was performed in Arlequin v3.5.2.2 to assess the proportion of genetic variation among and within populations, as well as among and within lineages.

### 4.3. Divergence Time and Demographic History

The mutation rate of the *psbA* gene sequence was estimated to be 0.08–0.12% per million years (Myr) based on methods from Hoarau et al. [78] and Uwai et al. [79]. The sequence divergence (*p*-distance) between *S. hemiphyllum* var. *chinense* and *Fucus vesiculosus* for the *psbA* sequence was calculated as 5.54% using data from GenBank (GenBank: MT800998, DQ307679). Consequently, the divergence time between these two algal species was estimated to be 46.17–69.25 million years ago (Mya) (i.e., 5.54%/0.12% Mya, 5.54%/0.08% Mya). Based on the experimental data and additional data from GenBank (GenBank: KM210510; MT845205; NC_007683; NC_016735), the sequence divergence between *S. hemiphyllum* var. *chinense* and *F. vesiculosus* for the *cox3*, *cox1*, and *rbcL* sequences was calculated as 18.08–18.22%, 17.14–17.27%, and 9.13–9.20%, respectively. Using the divergence time between the two algal species and the sequence divergence values for the three genes, the mutation rates were estimated as follows: the *cox3* sequence mutation rate was 0.26–0.39% per Myr (i.e., 18.08%/69.25 Myr, 18.22%/46.17 Myr), with an average of 0.33% per Myr; the *cox1* sequence mutation rate was 0.25–0.37% per Myr (i.e., 17.14%/69.25 Myr, 17.27%/46.17 Myr), with an average of 0.31% per Myr; and the *rbcL* sequence mutation rate was 0.13–0.20% per Myr (i.e., 9.13%/69.25 Myr, 9.20%/46.17 Myr), with an average of 0.17% per Myr.

The estimation of lineage divergence times was performed using the *cox3*, *cox1*, and *rbcL* sequence data from all individuals in the BEAST v1.10.4 software package [80]. First, the path sampling/stepping-stone sampling was used to perform marginal likelihood estimation (MLE) for five tree prior models (constant size, exponential growth, logistic growth, expansion growth, and Bayesian skyline). Based on the MLE results, the Bayesian skyline model was identified as the optimal tree prior. Next, a strict clock model and the estimated mutation rates for each sequence were applied, with the Bayesian skyline model selected as the tree prior. The number of groups was set to 10, the skyline model was set to piecewise-linear, and the tree model was set to UPGMA starting tree. The skyline.popSize prior parameter was configured with a lognormal prior distribution (initial value: 0.2, mean: 0.2, standard deviation: 0.1, offset: 0.0). The weight of the skyline.groupSize operator was adjusted to 100. The MCMC chain length was set to 100 million steps, with parameters logged every 10,000 steps. After the run, the log file was examined using Tracer v1.7.2 [81] to ensure convergence, with a burn-in of 10 million steps and effective sample sizes (ESSs) exceeding 200 for all parameters, confirming the reliability of the results. The tree file was imported into TreeAnnotator v1.10.4, with a burn-in of 10 million steps, to generate a maximum clade credibility tree. The calibrated gene tree was visualized using FigTree v1.4.4.

To infer the demographic history of different lineages, we employed neutrality tests, mismatch distribution analysis [82], and Bayesian skyline reconstruction [83]. Neutrality tests and mismatch distribution analyses were conducted using *cox3*–*cox1*–*rbcL* concatenated sequence data, while Bayesian skyline reconstruction was performed using individual *cox3*, *cox1*, and *rbcL* sequence data. The neutrality tests, including Tajima’s *D* [84] and Fu’s *F*_S_ [85], were calculated using the software Arlequin v3.5.2.2, with the number of simulated samples set to 10,000. Mismatch distribution analysis was also conducted in Arlequin v3.5.2.2. We first compared the sudden expansion model and the spatial expansion model using 10,000 bootstrap replicates, and the results indicated that the spatial expansion model provided a better fit to the data. For the spatial expansion model, we plotted the results and analyzed the *p*-values of the sum of squared deviation (*SSD*) and Harpending’s raggedness index (*Rag.*). Bayesian skyline reconstruction was performed using BEAST v1.10.4. The molecular clock, mutation rates, tree priors, prior parameters, and operator settings were consistent with those used for estimating lineage divergence times. The MCMC chain length was set to 25 million steps, with parameters logged every 2500 steps. The results were examined using Tracer v1.7.2 to ensure convergence, with ESS greater than 200 for all parameters at a burn-in of 2.5 million steps. Converged results were used to generate the Bayesian skyline plot in Tracer v1.7.2.

## 5. Conclusions

In this study, we conducted a phylogeographic analysis of *S. hemiphyllum* var. *chinense* along the coast of China, revealing the genetic pattern of this species and inferring the causes of its formation. We demonstrated the feasibility of using combined plastid and mitochondrial gene sequences as molecular markers in this research. Significant genetic structure differences were observed among 15 populations covering most of the distribution range of this species, which we attribute to colonization by ancestors from different sources and limited gene flow among populations. Two distinct lineages, which exhibit a north-south geographical distribution with a mixed zone in the southern Fujian-eastern Guangdong coastal region, are inferred to have diverged during the Middle to Late Pleistocene due to the isolation of the East China Sea and South China Sea during glacial periods. The northern lineage further exhibited sub-lineage differentiation. Demographic history inference suggested that the southern lineage underwent expansion following the end of the LGM, while the northern lineage remained stable. The southern Fujian–eastern Guangdong region, characterized by high genetic diversity, may have served as a glacial refugium or a contact zone for the post-glacial recolonization of the two lineages. Global warming may lead to range contraction and reduced genetic diversity in this species. The high genetic diversity area should be prioritized as a key region for species conservation. Our study enhances the understanding of the phylogeographic pattern of *S. hemiphyllum* var. *chinense*. Future research involving broader specimens sampling and more in-depth genetic information from both organellar and nuclear genomes will contribute to a clearer understanding of the current genetic structure, formation causes, and future trends of this species, as well as provide more background information for the development of species conservation strategies.

## Figures and Tables

**Table 1:** Genetic diversity indices of 15 populations based on the *cox3*–*cox1*–*rbcL* concatenated sequences.

Sampling Site	Code	Coordinates	*N*_h_/*N*_eh_	*H*_d_	*π* (×10^−2^)
Nanji Isl., Wenzhou	NJ	27.442 N, 121.066 E	2/1	0.517 ± 0.024	0.014 ± 0.001
Xiaozuo, Huian	XZ	24.959 N, 119.024 E	1/0	0	0
Liuao, Zhangpu	ZP	23.915 N, 117.774 E	3/0	0.384 ± 0.093	0.021 ± 0.005
Nanyu Isl., Dongshan	DS	23.722 N, 117.524 E	5/2	0.639 ± 0.052	0.034 ± 0.004
Dingpeng Isl., Nanao	DP	23.287 N, 117.301 E	2/1	0.331 ± 0.089	0.009 ± 0.002
Shenao Bay, Nanao	NA	23.482 N, 117.110 E	2/1	0.405 ± 0.078	0.011 ± 0.002
Pingyu Isl., Nanao	PY	23.332 N, 117.079 E	5/2	0.669 ± 0.074	0.042 ± 0.007
Keniaowei, Huilai	KN	23.018 N, 116.569 E	4/2	0.563 ± 0.072	0.046 ± 0.006
Zishen, Huilai	HL	22.966 N, 116.517 E	2/1	0.239 ± 0.092	0.006 ± 0.002
Zhelang, Shanwei	SW	22.656 N, 115.570 E	2/0	0.331 ± 0.089	0.009 ± 0.002
Yangmeikeng, Shenzhen	SZ	22.548 N, 114.574 E	3/2	0.515 ± 0.087	0.015 ± 0.003
Wailingding Isl., Zhuhai	WL	22.106 N, 114.022 E	1/0	0	0
Naozhou Isl., Zhanjiang	NZ	20.917 N, 110.637 E	4/3	0.251 ± 0.102	0.009 ± 0.004
Mulan Bay, Wenchang	ML	20.149 N, 110.690 E	2/1	0.370 ± 0.084	0.010 ± 0.002
Sitang, Xuwen	XW	20.230 N, 110.149 E	1/0	0	0
Total			20/16	0.692 ± 0.020	0.042 ± 0.001

*N*_h_: number of haplotypes, *N*_eh_: number of endemic haplotypes, *H*_d_: haplotype diversity, *π*: nucleotide diversity.

**Table 2:** Hierarchical analysis of molecular variance (AMOVA) in 15 populations and 2 lineages based on different sequences.

	Among Populations/Lineages		Within Populations/Lineages		*Φ*_ST_
	d.f.	% var	d.f.	% var	
Populations					
*rbcL*	14	10.34	435	89.66	0.10345 ***
*cox3*	14	77.29	435	22.71	0.77293 ***
*cox1*	14	62.87	435	37.13	0.62867 ***
*cox3*–*cox1*–*rbcL*	14	66.25	435	33.75	0.66252 ***
Lineages					
*cox3*–*cox1*–*rbcL*	1	79.22	448	20.78	0.79218 ***

d.f.: degree of freedom; % var: percentage of variation; *Φ*_ST_: fixation index; ***: *p* < 0.001.

**Table 3:** Neutrality tests of two lineages based on the *cox3*–*cox1*–*rbcL* concatenated sequence.

	Tajima’s *D*	Fu’s *F*_S_
Lineage N	−0.47261 (*p* = 0.31860)	−1.64597 (*p* = 0.25790)
Lineage S	−1.90073 (*p* = 0.00090)	−13.79697 (*p* = 0.00000)

## Data Availability

The original contributions presented in this study are included in the article/Supplementary Material. Further inquiries can be directed to the corresponding author.

## Supplementary Materials

The following supporting information can be downloaded at https://www.mdpi.com/article/10.3390/plants14091269/s1, Table S1: The distribution of ITS ribotypes; Table S2: Genetic diversity information of *rbcL* sequences from 15 populations; Table S3: Genetic diversity information of *cox3* sequences from 15 populations; Table S4: Genetic diversity information of *cox1* sequences from 15 populations; Table S5: Haplotype distribution of *cox1* sequences from 15 populations; Table S6: Haplotype distribution of *cox3*–*cox1*–*rbcL* concatenated sequences from 15 populations; Table S7: Pairwise genetic distance between 15 populations based on *cox3*–*cox1*–*rbcL* concatenated sequence; Table S8: Pairwise *F*_ST_ values between 15 populations based on *cox3*–*cox1*–*rbcL* concatenated sequence; Table S9: Geographical distances between 15 populations; Figure S1: Phylogenetic tree for ITS haplotypes. The 50% majority rule consensus tree. The nodes which bootstrap supports greater than 60 for ML tree and posterior probabilities greater than 0.9 for BI tree are labeled; Figure S2: Geographical distribution and frequency of *rbcL*, *cox3*, and *cox1* haplotypes. Pie chart denotes the proportion of haplotypes present in each population; Figure S3: Phylogenetic tree for *cox1* haplotypes. The 50% majority rule consensus tree. The nodes which bootstrap supports greater than 50 for ML tree and posterior probabilities greater than 0.7 for BI tree are labeled.
