# Supplementary material for: Phylogeographic Pattern of Sargassum hemiphyllum var. chinense (Phaeophyceae, Ochrophyta) in Chinese Coastal Waters
Source: Plants (Basel). 2025 Apr 22;14(9):1269. doi: 10.3390/plants14091269 (PMC12074080; doi:10.3390/plants14091269)
Supplement: Supplementary file 1 [file plants-14-01269-s001.zip › plants-3571599-supplementary.pdf]

Supplementary materials

Table S1 The distribution of ITS ribotypes

Population	N	N_m	N_r	Ribotypes
NJ	20	10	2	I-H1(10), I-H1/I-H2(10),
XZ	20	0	1	I-H1(20)
ZP	20	16	4	I-H1(4), I-H1/I-H2(14), I-H1/I-H3(1), I-H2/I-H4(1)
DS	20	0	1	I-H1(20)
DP	20	7	4	I-H1(13), I-H1/I-H2(1), I-H1/I-H3(3), I-H1/I-H5(3)
NA	20	0	1	I-H1(20)
PY	20	0	1	I-H1(20)
KN	20	11	2	I-H1(9), I-H1/I-H6(11)
HL	20	0	1	I-H1(20)
SW	20	3	6	I-H1(16), I-H1/I-H7(1), I-H1/I-H8(1), I-H3/I-H9(1), I-H10(1)
SZ	20	0	1	I-H1(20)
WL	20	1	2	I-H1(19), I-H1/I-H11(1)
NZ	20	1	2	I-H1(19), I-H1/I-H11(1)
ML	20	10	2	I-H1(10), I-H1/I-H2(10)
XW	20	5	3	I-H1/I-H12(3), I-H12(15), I-H12/I-H13(2)
Total	300	64	13	I-H1(220), I-H10(1), I-H12(15), I-H1/I-H2(35), I-H1/I-H3(4), I-H1/I-H5(3), I-H1/I-H6(11), I-H1/I-H7(1), I-H1/I-H8(1), I-H1/I-H11(2), I-H1/I-H12(3), I-H2/I-H4(1), I-H3/I-H9(1), I-H12/I-H13(2)

N: number of samples, N_m: number of multi-ribotypes samples, N_r: number of ribotypes. The numbers in parentheses represent the number of samples of the ribotype or the ribotype combination.

Table S2 Genetic diversity information of *rbcL* sequences from 15 populations

Population	N_{sv}	N_{pi}	N_h	N_{eh}	Number of haplotype samples					H_d	π ($\times10^{-2}$)
					r-H1	r-H2	r-H3	r-H4	r-H5		
NJ	0	0	1	0	30					0	0
XZ	0	0	1	0	30					0	0
ZP	0	0	1	0	30					0	0
DS	0	1	2	1	28	2				0.129±0.079	0.009±0.005
DP	0	1	2	1	24		6			0.331±0.089	0.023±0.006
NA	0	0	1	0	30					0	0
PY	0	0	1	0	30					0	0
KN	0	0	1	0	30					0	0

HL	0	0	1	0	30					0	0
SW	0	0	1	0	30					0	0
SZ	0	0	1	0	30					0	0
WL	0	0	1	0	30					0	0
NZ	0	2	3	2	26			2	2	0.248±0.100	0.018±0.007
ML	0	0	1	0	30					0	0
XW	0	0	1	0	30					0	0
Total	0	4	5	4	438	2	6	2	2	0.053±0.015	0.004±0.001

N_{sv}: Number of singleton variable sites, N_{pi}: Number of parsimony informative sites, N_h: Number of haplotypes, N_{eh}: Number of endemic haplotypes, H_d: Haplotype diversity, π: Nucleotide diversity

Table S3 Genetic diversity information of *cox3* sequences from 15 populations

Population	N_{sv}	N_{pi}	N_h	N_{eh}	Number of haplotype samples				H_d	π ($\times10^{-2}$)
					c3-H1	c3-H2	c3-H3	c3-H4		
NJ	0	1	2	1	15	15			0.517±0.024	0.070±0.003
XZ	0	0	1	0		30			0	0
ZP	0	1	2	0		7	23		0.370±0.084	0.050±0.011
DS	1	0	2	0		29	1		0.067±0.061	0.009±0.008
DP	0	0	1	0		30			0	0
NA	0	0	1	0		30			0	0
PY	0	2	3	1		7	20	3	0.508±0.085	0.075±0.015
KN	0	1	2	0		18	12		0.497±0.043	0.067±0.006
HL	0	0	1	0			30		0	0
SW	0	0	1	0			30		0	0
SZ	0	0	1	0			30		0	0
WL	0	0	1	0			30		0	0
NZ	0	0	1	0			30		0	0
ML	0	0	1	0			30		0	0
XW	0	0	1	0			30		0	0
Total	0	3	4	2	15	166	266	3	0.514±0.013	0.076±0.003

N_{sv}: Number of singleton variable sites, N_{pi}: Number of parsimony informative sites, N_h: Number of haplotypes, N_{eh}: Number of endemic haplotypes, H_d: Haplotype diversity, π: Nucleotide diversity

Table S4 Genetic diversity information of *cox1* sequences from 15 populations

Population	N_{sv}	N_{pi}	N_h	N_{eh}	H_d	π ($\times10^{-2}$)
NJ	0	0	1	0	0	0
XZ	0	0	1	0	0	0
ZP	1	1	3	0	0.384±0.093	0.028±0.008
DS	1	2	4	1	0.579±0.047	0.069±0.005
DP	0	0	1	0	0	0
NA	0	1	2	1	0.405±0.078	0.025±0.005
PY	1	3	4	1	0.559±0.086	0.066±0.013

KN	1	3	4	2	0.563±0.072	0.079±0.011
HL	0	1	2	1	0.239±0.092	0.015±0.006
SW	0	1	2	0	0.331±0.089	0.021±0.006
SZ	0	2	3	2	0.515±0.087	0.036±0.007
WL	0	0	1	0	0	0
NZ	1	0	2	1	0.067±0.061	0.004±0.004
ML	0	1	2	1	0.370±0.084	0.023±0.005
XW	0	0	1	0	0	0
Total	3	10	14	10	0.657±0.018	0.063±0.003

N_{sv}: Number of singleton variable sites, N_{pi}: Number of parsimony informative sites, N_h: Number of haplotypes, N_{eh}: Number of endemic haplotypes, H_d: Haplotype diversity, π: Nucleotide diversity

Table S5 Haplotypes distribution of *cox1* sequences from 15 populations

Population	Number of haplotype samples								
	c1-H1	c1-H2	c1-H3	c1-H4	c1-H5	c1-H6	c1-H7	c1-H8	c1-H9
NJ	30								
XZ	30								
ZP	6	23	1						
DS	15	1	1	13					
DP	30								
NA			22		8				
PY		19	6			4	1		
KN		9	18					2	1
HL		26							
SW		24				6			
SZ		20							
WL		30							
NZ		29							
ML		23							
XW		30							
Total	111	234	48	13	8	10	1	2	1

(Continued)

Population	Number of haplotype samples				
	c1-H10	c1-H11	c1-H12	c1-H13	c1-H14
NJ					
XZ					
ZP					
DS					
DP					
NA					
PY					
KN					

HL	4				
SW					
SZ		6	4		
WL					
NZ				1	
ML					7
XW					
Total	4	6	4	1	7

Table S6 Haplotypes distribution of *cox3–cox1–rbcL* concatenated sequences from 15 populations

Population	Number of haplotype samples												
	H1	H2	H3	H4	H5	H6	H7	H8	H9	H10	H11	H12	H13
NJ	15	15											
XZ		30											
ZP		6	23	1									
DS		13	1	1	13	2							
DP		24					6						
NA				22				8					
PY			16	6					4	1	3		
KN			9	18								2	1
HL			26										
SW			24						6				
SZ			20										
WL			30										
NZ			26										
ML			23										
XW			30										
Total	15	88	228	48	13	2	6	8	10	1	3	2	1

(Continued)

Population	Number of haplotype samples						
	H14	H15	H16	H17	H18	H19	H20
NJ							
XZ							
ZP							
DS							
DP							
NA							
PY							
KN							
HL	4						
SW							
SZ		6	4				

WL							
NZ				2	1	1	
ML							7
XW							
Total	4	6	4	2	1	1	7

Table S7 Pairwise genetic distance between 15 populations based on *cox3–cox1–rbcL* concatenated sequence

（$\times10^{-4}$）

	NJ	XZ	ZP	DS	DP	NA	PY	KN	HL	SW	SZ
NJ		1.244	2.943	2.122	1.359	3.032	3.140	2.530	3.730	3.805	3.685
XZ	1.318		2.770	1.730	0.607	2.645	2.907	2.167	3.635	3.660	3.566
ZP	5.454	4.133		3.146	2.804	3.838	1.546	2.590	0.932	1.015	0.975
DS	4.043	2.724	6.506		1.864	2.070	3.231	2.336	3.840	3.902	3.799
DP	1.846	0.527	4.661	3.252		2.662	2.942	2.217	3.652	3.683	3.569
NA	4.658	3.339	7.298	3.603	3.866		3.539	1.981	4.577	4.588	4.529
PY	6.685	5.364	3.253	7.246	5.892	7.475		2.591	1.213	1.343	1.242
KN	5.364	4.044	4.836	5.152	4.572	4.222	5.434		2.777	2.814	2.729
HL	6.950	5.629	1.671	8.002	6.157	8.971	2.901	5.452		0.627	0.704
SW	7.125	5.804	1.846	8.177	6.332	9.146	2.936	5.627	0.879		0.789
SZ	7.478	6.156	2.198	8.530	6.685	9.499	3.429	5.979	1.230	1.406	
WL	6.598	5.276	1.319	7.650	5.805	8.618	2.549	5.100	0.352	0.527	0.879
NZ	7.038	5.716	1.759	8.090	6.245	9.058	2.989	5.540	0.791	0.967	1.318
ML	7.213	5.892	1.934	8.265	6.420	9.234	3.165	5.715	0.967	1.142	1.494
XW	6.598	5.276	1.319	7.650	5.805	8.618	2.549	5.100	0.352	0.527	0.879

(Continued)

	WL	NZ	ML	XW
NJ	3.713	3.725	3.751	3.713
XZ	3.605	3.616	3.666	3.605
ZP	0.850	0.889	1.049	0.850
DS	3.824	3.855	3.887	3.824
DP	3.624	3.644	3.674	3.624
NA	4.549	4.570	4.595	4.549
PY	1.154	1.188	1.343	1.154
KN	2.740	2.767	2.814	2.740
HL	0.351	0.455	0.701	0.351
SW	0.530	0.601	0.802	0.530
SZ	0.619	0.676	0.870	0.619
WL		0.244	0.597	0.000
NZ	0.439		0.635	0.244
ML	0.615	1.054		0.597
XW	0.000	0.439	0.615	

Pairwise genetic distance between populations on lower left. Standard error (S. E.) on upper right.

Table S8 Pairwise F_{ST} values between 15 populations based on *cox3–cox1–rbcL* concatenated sequence

	NJ	XZ	ZP	DS	DP	NA	PY	KN
XZ	0.48276***							
ZP	0.70432***	0.74258***						
DS	0.41772***	0.38087***	0.57833***					
DP	0.40828***	0.17241*	0.69248***	0.35518***				
NA	0.74432***	0.83945***	0.79036***	0.35479***	0.75099***			
PY	0.60912***	0.60558***	0.01428^{ns}	0.49092***	0.58235***	0.66395***		
KN	0.45571***	0.40826***	0.28389***	0.22949***	0.39835***	0.35751***	0.18277**	
HL	0.87724***	0.94924***	0.19184***	0.75107***	0.89224***	0.91628***	0.15975***	0.51281***
SW	0.86367***	0.93166***	0.19394***	0.74295***	0.87684***	0.90585***	0.12768**	0.50447***
SZ	0.83720***	0.89036***	0.19538***	0.72478***	0.84433***	0.88174***	0.17269***	0.49309***
WL	0.90988***	1***	0.19437*	0.77270***	0.93240***	0.94431***	0.15258**	0.52931***
NZ	0.86357***	0.93029***	0.16892***	0.74116***	0.87613***	0.90512***	0.14930***	0.50111***
ML	0.85914***	0.92472***	0.20408***	0.74060***	0.87137***	0.90193***	0.17477***	0.50417***
XW	0.90988***	1***	0.19437*	0.77270***	0.93240***	0.94431***	0.15258**	0.52931***

(Continued)

	HL	SW	SZ	WL	NZ	ML
XZ						
ZP						
DS						
DP						
NA						
PY						
KN						
HL						
SW	0.14709**					
SZ	0.14045***	0.16099***				
WL	0.10345^{ns}	0.17241*	0.14203**			
NZ	0.06874**	0.11037**	0.11306***	0.02610^{ns}		
ML	0.17150**	0.20190***	0.17682***	0.20690**	0.13593***	
XW	0.10345^{ns}	0.17241*	0.14203**	0^{ns}	0.02610^{ns}	0.20690*

*：$p < 0.05$；**：$p < 0.01$；***：$p < 0.001$；^{ns}：统计差异不显著($p > 0.05$)

*: $p < 0.05$; **: $p < 0.01$; ***: $p < 0.001$; ^{ns}: not significant ($p > 0.05$)

Table S9 Geographical distances between 15 populations

（×100 km）

	NJ	XZ	ZP	DS	DP	NA	PY	KN	HL
XZ	3.4617								
ZP	5.1515	1.7185							

DS	5.4833	2.0515	0.3335						
DP	5.9956	2.5489	0.8442	0.5311					
NA	5.958	2.5455	0.8315	0.4995	0.297				
PY	6.1034	2.6801	0.9614	0.6261	0.2408	0.1661			
KN	6.6883	3.3017	1.5846	1.2512	0.8148	0.756	0.628		
HL	6.7669	3.3799	1.6632	1.33	0.887	0.8351	0.7055	0.0793	
SW	7.6846	4.3523	2.6536	2.3243	1.9163	1.8249	1.7197	1.1014	1.0307
SZ	8.5151	5.268	3.6093	3.2909	2.9224	2.7995	2.7119	2.113	2.0471
WL	9.2686	6.0111	4.3497	4.0158	3.623	3.5193	3.4227	2.8083	2.7374
NZ	12.83	9.7003	8.0661	7.7467	7.3707	7.2555	7.166	6.5561	6.4859
ML	13.3118	10.098	8.4239	8.0957	7.6837	7.5972	7.4939	6.8712	6.7973
XW	13.6961	10.5239	8.8666	8.5433	8.1451	8.0473	7.9492	7.3302	7.2577

(Continued)

	SZ	WL	NZ	ML
XZ				
ZP				
DS				
DP				
NA				
PY				
KN				
HL				
SW				
SZ				
WL	0.7519			
NZ	4.4591	3.7486		
ML	4.8314	4.0879	0.8562	
XW	5.2609	4.5288	0.9155	0.5722

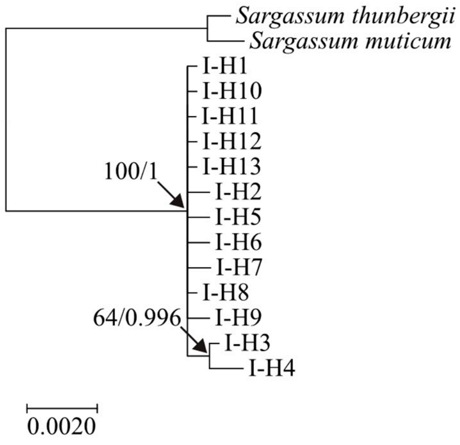

Figure S1 Phylogenetic tree for ITS haplotypes. The 50% majority rule consensus tree. The nodes which bootstrap supports greater than 60 for ML tree and posterior probabilities greater than 0.9 for BI tree are labeled.

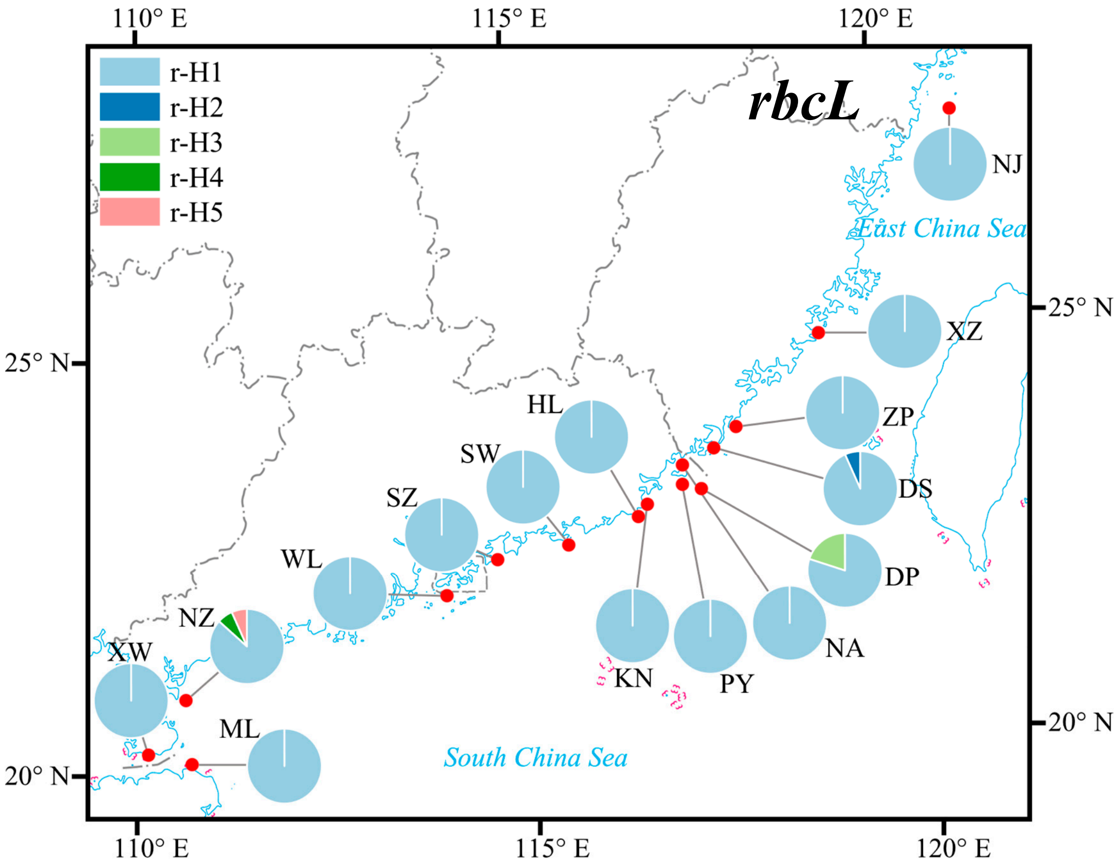

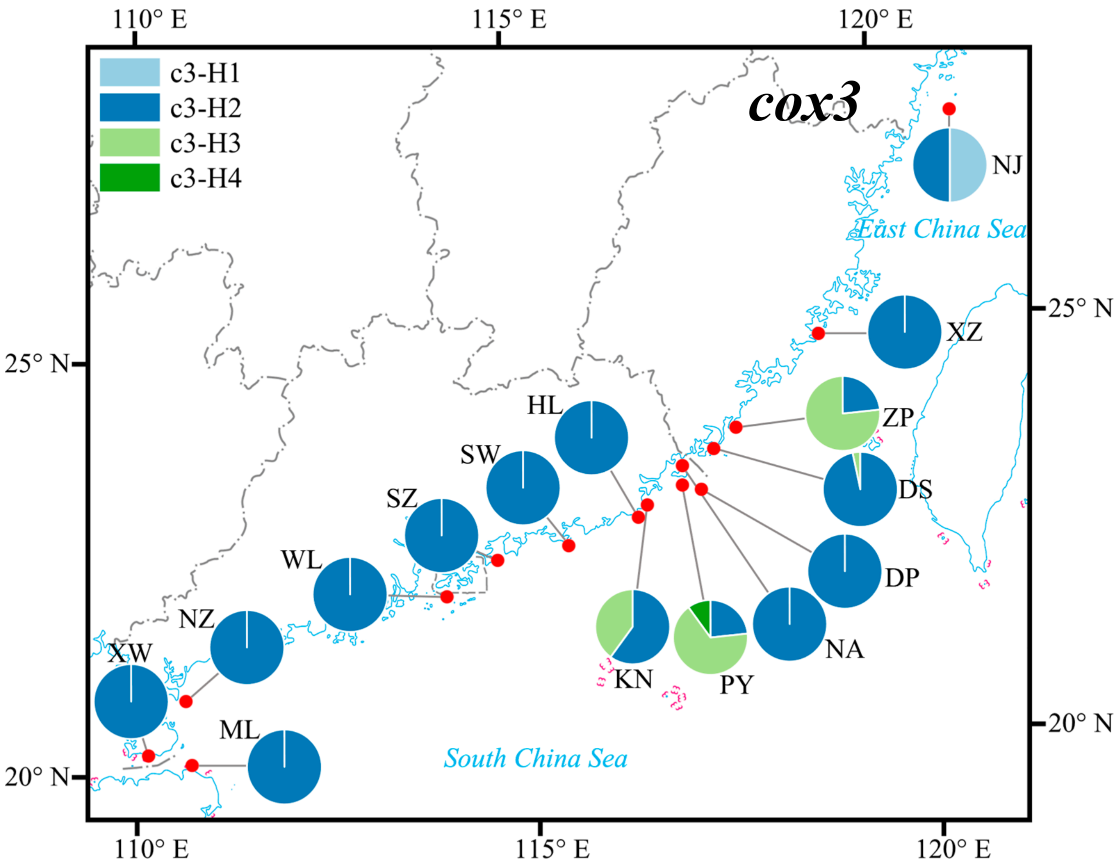

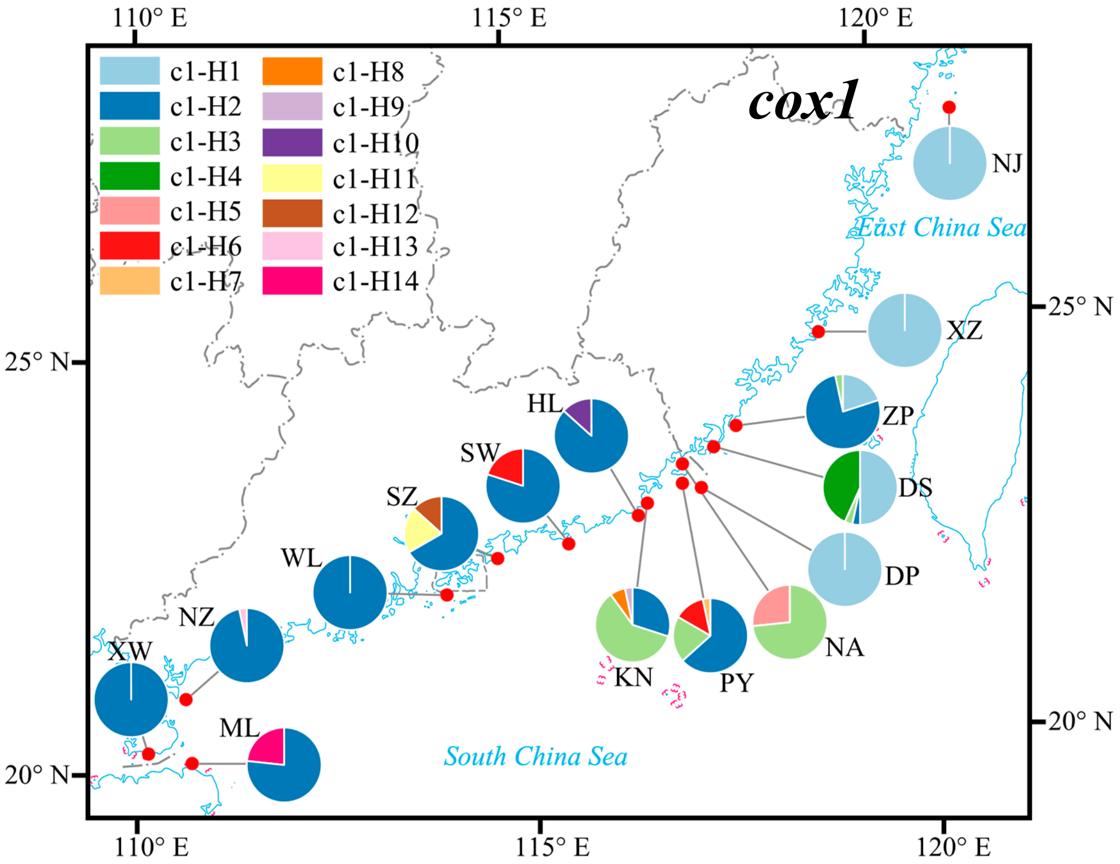

Figure S2 Geographical distribution and frequency of *rbcL*, *cox3* and *cox1* haplotypes. Pie chart denotes the proportion of haplotypes present in each population.

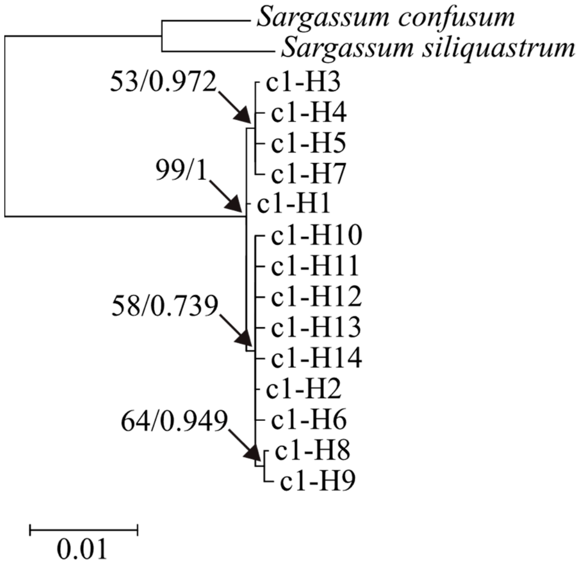

Figure S3 Phylogenetic tree for *cox1* haplotypes. The 50% majority rule consensus tree. The nodes which bootstrap supports greater than 50 for ML tree and posterior probabilities greater than 0.7 for BI tree are labeled.
